# The Economic Value of Reducing Adult Obesity in Greece: A Prevalence-Based Dynamic Modelling Study

**DOI:** 10.3390/healthcare14213578

**Published:** 2026-10-22

**Authors:** Panagiotis Papantoniou, Nikos Maniadakis, Tonia Vassilakou

**Affiliations:** Department of Public Health Policy, School of Public Health, University of West Attica, 196 Alexandras Avenue, 115 21 Athens, Greece; nmaniadakis@uniwa.gr (N.M.); tvasilakou@uniwa.gr (T.V.)

**Keywords:** obesity, economic burden, obesity prevalence, cost savings, Greece

## Abstract

**Highlights:**

**What are the main findings?**

- Adult obesity is projected to remain a substantial and increasing economic burden in Greece, with annual societal costs rising from approximately EUR 4.23 billion in 2025 to EUR 5.11 billion by 2055, driven predominantly by obesity-related comorbidities and productivity losses.
- Under a hypothetical 5% annual relative reduction in adult obesity prevalence, the model projected EUR 71.12 billion in cumulative gross costs avoided (EUR 29.25 billion discounted) over 2026–2055 compared with the status quo.

**What are the implications of the main findings?**

- The economic burden of obesity is driven primarily by downstream chronic disease and productivity losses rather than obesity-specific treatment expenditure, highlighting the value of public health policies before costly complications manifest and accumulate.
- The findings provide a framework for comparing the potential long-term economic consequences of alternative obesity-prevalence trajectories, but do not establish the feasibility, implementation costs or cost-effectiveness of specific obesity-reduction policies.

**Abstract:**

Background/Objectives: Obesity represents a major and growing economic challenge, yet evidence on its long-term economic burden and the potential value of sustained reductions in obesity prevalence remains limited in Greece. This study estimated the future economic burden of adult obesity in Greece and quantified the gross healthcare and societal costs avoided from alternative sustained reductions in obesity prevalence. Methods: A prevalence-based, top–down dynamic model was developed for the Greek adult population over 2026–2055. The analysis adopted a societal perspective and incorporated direct medical costs associated with obesity pharmacotherapy, bariatric surgery, and 36 obesity-related comorbidities, together with productivity losses from absenteeism and presenteeism. Adult population and obesity prevalence were projected using exponential smoothing, while population-attributable fractions (PAFs) linked obesity prevalence to comorbidity-related expenditure. The primary illustrative scenario assumed a hypothetical 5% annual relative reduction in adult obesity prevalence, rather than a 5% reduction in individual body weight. Potential gross costs avoided were estimated by comparing the projected obesity-attributable economic burden under this scenario with the corresponding burden under the status quo trajectory over the 2026–2055 modelling horizon. Costs were expressed in 2025 euros and discounted at 5% annually. Scenario, deterministic, and probabilistic sensitivity analyses were conducted. Results: Under the status quo, adult obesity prevalence was projected to increase from 29.24% in 2025 to 42.87% in 2055, while annual obesity-attributable costs increased from EUR 4.23 billion to EUR 5.11 billion. Direct medical costs accounted for 86.38% of cumulative expenditure. Under the 5% annual prevalence-reduction scenario, obesity prevalence declined to 6.28% by 2055, generating cumulative undiscounted gross costs avoided of EUR 71.12 billion and discounted gross costs avoided of EUR 29.25 billion. Probabilistic analysis produced discounted gross costs avoided of approximately EUR 27.85 billion, with a 95% uncertainty interval of EUR 23.83–31.61 billion. Conclusions: Adult obesity is projected to impose a substantial economic burden on Greece, with the findings illustrating the potential gross healthcare and societal costs avoided under hypothetical, sustained reductions in adult obesity prevalence, rather than the expected economic outcomes of specific interventions. These estimates are conditional on the assumed prevalence trajectories and do not establish the feasibility or cost-effectiveness of achieving such reductions.

## 1. Introduction

Obesity is a chronic, relapsing disease characterised by excess adiposity that adversely affects health [1]. It is commonly defined using body mass index (BMI), calculated as weight in kilograms divided by height in metres squared, with BMI ≥30 kg/m^2^ indicating obesity [1]. Obesity is widely recognised as a complex and multifactorial condition resulting from numerous interrelated metabolic, behavioural, socio-economic, environmental, psychosocial and genetic determinants [1].

The global burden of obesity has risen dramatically over recent decades, reaching pandemic proportions. In 2022, more than 890 million adults worldwide were living with obesity, more than double the number recorded in 1990 [1]. If current trends persist, global prevalence is projected to exceed one billion adults by 2030 [2,3]. Greece mirrors this concerning trajectory, with the World Health Organisation (WHO) estimating that approximately 28% of Greek adults, or roughly 2.4 million individuals, were living with obesity in 2022 [4]. In response to this growing burden, the Greek Ministry of Health recently introduced a national adult obesity action plan that allows eligible individuals with severe obesity or obesity with comorbidities (BMI ≥ 37–40 kg/m^2^) to access pharmacotherapy and lifestyle interventions at no cost [5].

Obesity is a well-established risk factor for a broad range of comorbidities, including cardiometabolic and cardiovascular diseases, musculoskeletal disorders, respiratory and gastrointestinal conditions, reproductive disorders, mental health conditions and several cancer types [6]. Individuals living with obesity face significantly higher mortality risk compared with those with normal BMI, with meta-analytic evidence suggesting an approximately 18% increase in all-cause mortality [7]. Global Burden of Disease (GBD) estimates attribute nearly five million premature deaths annually to elevated BMI [8]. In addition to increased mortality, obesity is associated with substantial reductions in life expectancy, with severe obesity linked to losses of up to 8–10 years [6], while consistently impairing health-related quality of life across physical, psychological and social domains [9,10].

The economic implications of obesity are equally substantial. Globally, the cost of overweight and obesity is projected to increase from approximately USD 2.47 trillion in 2025 to USD 4.32 trillion by 2035, corresponding to nearly 3% of global gross domestic product (GDP) [2]. National estimates for Greece show a similarly pronounced burden: a recent cost-of-illness (COI) study estimated that obesity imposed a total societal cost of EUR 4.92 billion in 2024, or 2.07% of national GDP, with obesity-related comorbidities accounting for most expenditures [11]. Future projections are similarly concerning: by 2050, the World Obesity Federation estimates that the economic burden of obesity in Greece could reach approximately EUR 7.7 billion, with indirect costs accounting for most of the total costs [2].

Sustained weight loss, irrespective of baseline BMI, is associated with clinically meaningful health benefits. Even modest reductions in body weight (3–5%) can improve cardiometabolic risk factors and other obesity-related outcomes, while greater weight loss is associated with progressively larger benefits [12]. Longitudinal real-world analyses have demonstrated that weight reduction is associated with clinically relevant reductions in the incidence of multiple comorbidities compared with weight stability [13]. Weight loss has also been shown to improve health-related quality of life, particularly across physical and functional domains [14]. These clinical improvements may translate into substantial economic benefits, with several modelling studies showing that sustained weight loss reduces healthcare expenditure, particularly among individuals with obesity-related comorbidities [15,16,17]. Simulation analyses from Europe and the United States further suggest that population-level weight reduction could generate considerable healthcare savings over both short- and long-term horizons [18,19,20]. Moreover, weight loss may yield indirect economic benefits by improving workplace productivity through reductions in absenteeism and presenteeism [21,22].

Despite this growing body of evidence, important gaps remain in the international scholarship. Many economic studies of weight loss focus on a limited subset of comorbidities, most commonly diabetes or cardiovascular disease and therefore fail to capture the broader spectrum of obesity-related conditions [13,15,16,17,18,19]. Additionally, studies often exclude direct costs associated with obesity management, including pharmacotherapy and bariatric surgery. Indirect costs from productivity losses are also incorporated inconsistently, and relatively few studies adopt a comprehensive societal perspective [21,22]. Most existing analyses focus on selected patient subgroups rather than the overall population living with obesity, limiting their relevance for informing population-level policy decisions [18,19,20].

Furthermore, many modelling approaches rely on epidemiological risk engines or parameter estimates from other countries, particularly the United Kingdom, which may not fully generalise to national healthcare settings [13]. Existing models also frequently assume static populations or isolated prevalence changes, without accounting for demographic shifts such as population ageing and decline, factors relevant in Western countries, including Greece. Finally, uncertainty surrounding key epidemiological and economic parameters is often insufficiently explored, thereby reducing confidence in long-term projections.

These limitations are especially relevant in Greece. Despite the high and increasing prevalence of obesity and its associated economic burden, no comprehensive analysis has quantified the long-term economic value of sustained obesity reduction across the entire adult population in Greece. Such evidence is essential to allocate scarce healthcare resources efficiently and evaluate the potential economic benefits of current and future obesity-related public health policies.

This study addresses this evidence gap using a dynamic, prevalence-based modelling framework to estimate the long-term economic burden of adult obesity in Greece and quantify potential healthcare and societal cost savings from sustained reductions in obesity prevalence over a 30-year time horizon.

## 2. Materials and Methods

### 2.1. Study Design

This study employed a prevalence-based dynamic modelling framework to estimate the long-term economic impact of reducing adult obesity in Greece. The model was developed in Microsoft Excel (version 16.0; Microsoft Corporation, Redmond, WA, USA)and used a top–down COI approach, using aggregate national demographic, epidemiological, and economic data to quantify the current and projected burden of obesity and the potential cost savings from sustained reductions in obesity prevalence. By integrating population projections, epidemiological trends and disease-specific cost data, the model generated annual estimates of direct and indirect costs attributable to obesity and compared these across alternative future scenarios.

The modelling framework was specifically designed to inform population-level policy decisions. Unlike individual-level simulation models or cohort-based risk-engine approaches commonly used in economic evaluations of weight loss [15,18,19,20], which typically simulate hypothetical patient cohorts and frequently rely on risk equations derived from external populations, the present analysis estimated the burden of obesity across the entire adult population of Greece. In addition, whereas many previous studies have focused on a limited subset of obesity-related comorbidities or relatively short analytic horizons [17,20], the present model incorporated a broad range of obesity-related conditions and projected outcomes over a 30-year horizon while explicitly accounting for demographic changes. This feature is particularly relevant in Greece, where population ageing and demographic decline are expected to influence future healthcare demand and expenditure substantially.

The analysis followed a prevalence-based methodology, applying annual obesity prevalence estimates to projected adult population counts to estimate the number of individuals living with obesity each year. We then used a top–down costing approach to estimate economic burden by applying disease-specific attribution factors and unit cost estimates to these population projections. Given the established causal relationship between obesity and multiple chronic diseases, we used the PAF methodology to estimate the proportion of each comorbidity directly attributable to obesity [23,24,25,26,27,28]. The PAF approach is widely used in obesity-related COI studies to estimate the proportion of population-level disease burden statistically attributable to obesity under a specified exposure distribution [28,29,30]. Accordingly, we recalculated annual PAFs under each alternative obesity-prevalence trajectory to estimate the corresponding population-level obesity-attributable comorbidity burden and associated economic outcomes. Importantly, this approach does not imply that individuals moving below the BMI threshold for obesity immediately experience remission of established disease or cease to incur the corresponding healthcare costs. Rather, the scenario comparisons estimate differences in population-level obesity-attributable burden under alternative obesity-prevalence trajectories. The model was not designed as an individual-level disease-history simulation and therefore does not explicitly model disease onset, remission, progression, recurrent events or residual risk following changes in BMI. We also estimated the resulting changes in annual costs contemporaneously within each modelled year. The framework did not explicitly simulate disease latency, incidence, progression, or remission, nor did it incorporate disease-specific mortality, competing risks, or transitions between concurrent comorbidity states. Consequently, the model estimated comparative annual attributable economic burden under alternative obesity-prevalence trajectories, rather than the timing of realised healthcare savings following adult obesity reduction.

To estimate the economic burden comprehensively, we included both direct medical and indirect costs. Direct costs included obesity management interventions and obesity-related comorbidities, while indirect costs included productivity losses from absenteeism and presenteeism. We derived aggregate epidemiological, morbidity, and cost data from national and international sources in accordance with standard COI methodology.

We expressed all monetary values in 2025 euros. We inflated historical cost inputs to 2025 price levels using the annual average Consumer Price Index (CPI) published by the Hellenic Statistical Authority (ELSTAT), ensuring comparability across time [31]. Costs derived from official reimbursement sources, including national price bulletins and diagnosis-related group tariffs, were not inflated when they already reflected current reimbursement values. The mathematical specifications underlying the principal model components and economic outcomes are provided in Supplementary Table S1.

The model evaluated outcomes over a 30-year time horizon (2026–2055), with 2025 serving as the baseline year for all epidemiological and economic inputs. This time horizon was selected to adequately capture the long-term economic consequences of current obesity trends while remaining relevant to contemporary healthcare policy planning. We discounted future costs and savings at an annual rate of 5%, consistent with commonly applied discounting practices in Greek health-economic evaluations [11,32,33,34,35].

Model calculations followed a structured annual sequence: (1) projection of total and adult population size; (2) application of projected obesity prevalence rates; (3) estimation of the number of adults living with obesity; (4) estimation of the number of individuals eligible for pharmacotherapy and bariatric surgery; (5) estimation of obesity-attributable comorbidity burden using PAFs; (6) calculation of direct and indirect costs; and (7) aggregation of total annual economic burden. Two principal scenarios were evaluated. Under the future (status-quo) scenario, obesity prevalence followed projected trends. In the obesity prevalence reduction scenario, obesity prevalence declined annually to meet predefined reduction targets (1–20%). For each projected year, the difference in total costs between scenarios represented the economic benefit associated with obesity reduction, while cumulative discounted savings were estimated over the entire study horizon. A comprehensive overview of the principal epidemiological, clinical and economic input parameters, together with their methodological sources and uncertainty assumptions, is presented in Supplementary Table S2.

This modelling framework was selected because it provides transparent, reproducible and policy-relevant estimates of the national burden attributable to obesity and the potential economic returns associated with sustained obesity-reduction strategies. By combining a population-wide perspective, a long-term time horizon, and comprehensive inclusion of direct and indirect costs, the model offers a pragmatic, robust framework to inform national public health policy and long-term resource allocation decisions.

**Figure 1:**
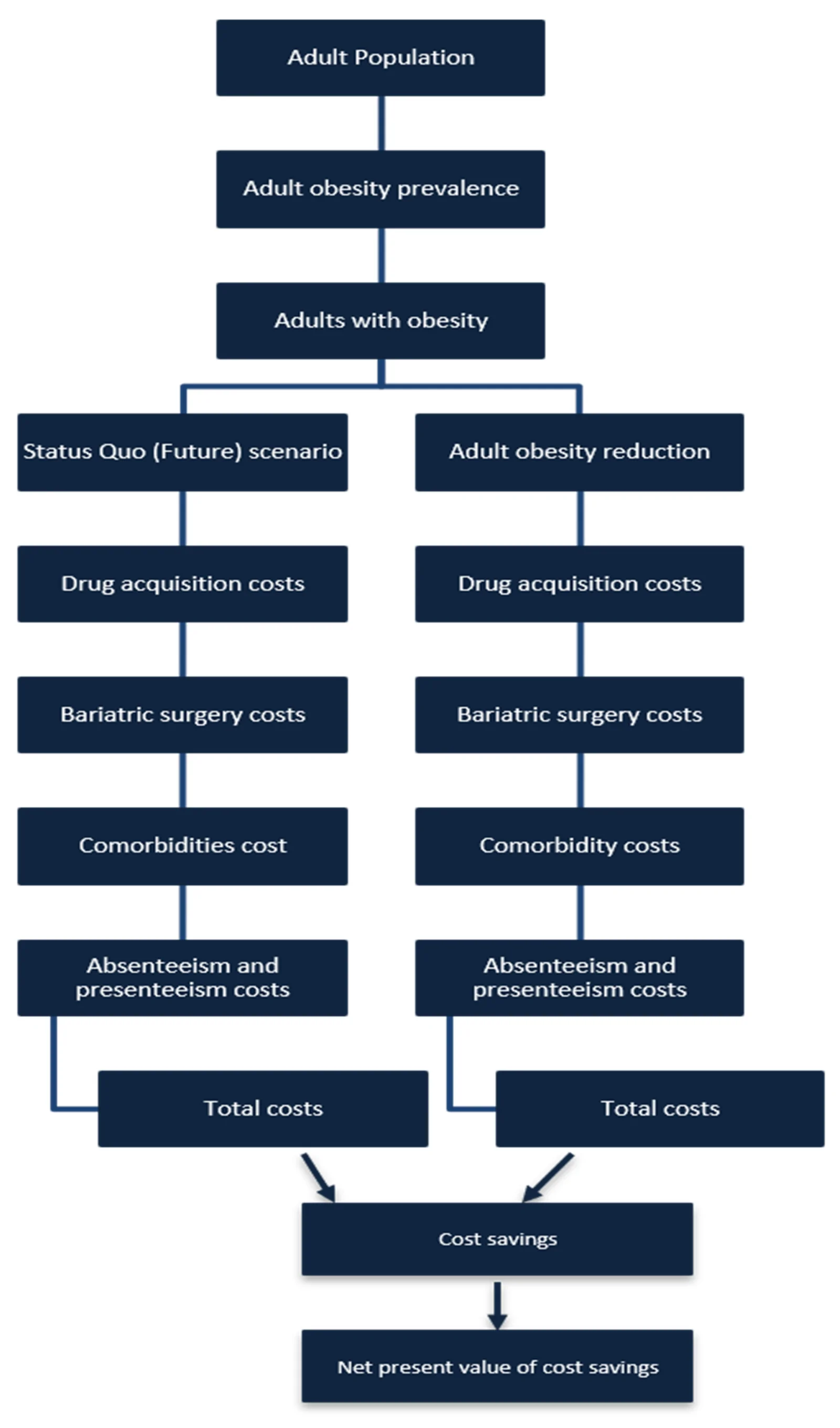
Schematic overview of the obesity forecasting model.

The model’s dynamic component involves annual updates to adult population size, obesity prevalence, obesity-attributable fractions, and associated economic outcomes over the projection horizon. It does not represent an individual-level dynamic disease model. We treated disease-specific prevalence estimates, relative risks, and unit costs as fixed parameters unless varied in sensitivity analyses, and the framework did not explicitly simulate individual disease progression, treatment pathways, mortality, or transitions between clinical states. Figure 1 presents a schematic overview of the model structure.

### 2.2. Population and Demographic Projections

We obtained national population data from the Hellenic Statistical Authority (ELSTAT), including annual estimates of Greece’s adult population for 2001–2025 [36]. These data constituted the empirical basis for projecting demographic trends over the modelling horizon (2026–2055).

To generate future population projections, we used an exponential smoothing state-space model with error, trend, and seasonality components (ETS). This modelling approach is widely used in demographic and epidemiological research to estimate future values by assigning progressively greater weight to more recent observations while capturing underlying temporal trends [37,38,39,40]. In the present analysis, we incorporated a non-seasonal trend component into the ETS specification, which we considered appropriate for annual demographic data. We selected this approach because it is robust to structural changes, performs well in relatively short time series, and has been shown to suit long-term demographic and epidemiological forecasting [37,38,39,40].

A key advantage of the ETS framework is its capacity to generate prediction intervals around projected estimates [39,40]. We retained these intervals and incorporated them into sensitivity analyses to account for uncertainty in future population trajectories. In addition, the ETS methodology allows dynamic recalibration as new official population data become available, thereby ensuring that the modelling framework remains adaptable and policy-relevant over time [40].

Incorporating projected demographic changes was essential to estimate Greece’s future obesity burden accurately. Official ELSTAT data indicate that between 2011 and 2025, the total population declined by approximately 53,647 individuals annually [36]. At the same time, population ageing substantially shifted the demographic structure, with the proportion of adults increasing from approximately 80.8% in 2001 to 83.96% in 2025. These demographic dynamics are expected to directly influence both the number of individuals living with obesity and the future burden of obesity-related disease. Failing to account for these changes would likely bias estimates of future healthcare utilisation and societal costs.

### 2.3. Epidemiology of Adult Obesity and Forecasting

In this study, we defined obesity according to World Health Organisation (WHO) criteria as a body mass index (BMI) ≥ 30 kg/m^2^ among adults [1]. To identify nationally representative prevalence estimates for Greece, we conducted a structured review of epidemiological evidence using major scientific databases, including PubMed, Google Scholar, and Web of Science.

We retrieved annual adult obesity prevalence data from the WHO Global Health Observatory (GHO), which provides harmonised country-level prevalence estimates from 1990 onwards, along with corresponding confidence intervals [4]. We selected these data as the primary epidemiological source for the modelling framework, consistent with previous cost-of-illness studies on obesity in Greece, to ensure methodological consistency and comparability with existing evidence [11].

The WHO estimates synthesise multiple epidemiological data sources and apply standardised statistical modelling techniques to generate internally consistent and internationally comparable prevalence estimates over time [4]. This methodology minimises heterogeneity arising from differences in study design, sampling approaches and measurement techniques, while providing a coherent longitudinal time series suitable for long-term forecasting analyses. Consequently, we considered the WHO GHO dataset the most robust and internally consistent source for the present modelling study. The primary analysis used age-standardised adult obesity-prevalence estimates from the WHO Global Health Observatory to align with the original epidemiological forecasting framework and facilitate comparison with previously published estimates. Nevertheless, age-standardised prevalence reflects the prevalence that would be observed under a standard reference age distribution and is therefore not directly interchangeable with crude prevalence when estimating the absolute number of individuals living with obesity in the actual population. Applying age-standardised prevalence to projected adult population counts can introduce a denominator mismatch, particularly in the context of demographic ageing. For example, in 2024, the WHO reported an age-standardised adult obesity prevalence of 27.43% for Greece, compared with a crude prevalence of 33.7%. Although the former was retained as the primary modelling specification, its application to the actual adult population was recognised as a structural assumption rather than a direct estimate of national obesity case counts. To evaluate the influence of this assumption, an independent scenario analysis based on crude adult obesity prevalence was undertaken, as described in Section 2.10. The historical age-standardised obesity-prevalence estimates, annual forecasts and corresponding lower and upper forecast limits are provided in Supplementary Table S3 whilst the corresponding crude obesity-prevalence estimates are presented in Supplementary Table S4.

Annual obesity prevalence estimates for 1990–2024 formed the empirical basis for future projections. To maintain methodological consistency with the demographic forecasting framework, we projected future obesity prevalence for 2025–2055 using an exponential smoothing state-space model with error, trend, and seasonality components (ETS). Consistent with the demographic projections, the ETS specification incorporated a non-seasonal trend component to reflect the persistent long-term increase in obesity prevalence.

The ETS framework additionally generated confidence intervals around projected prevalence estimates. We retained these intervals and incorporated them into sensitivity analyses to account for uncertainty in future obesity trajectories. As with the population forecasting framework, the obesity projection model can be dynamically recalibrated as updated prevalence data become available, ensuring long-term adaptability and policy relevance.

We then applied projected obesity prevalence estimates to annual adult population projections to estimate the number of adults living with obesity each year of the modelling horizon. In the primary base-case analysis, we combined obesity prevalence projections with dynamically changing population estimates to capture the joint effects of epidemiological and demographic change.

We assessed forecast plausibility by checking whether projected obesity-prevalence trajectories are consistent with historical WHO estimates and by comparing long-term projections with independently published epidemiological forecasts, as discussed in the Results. These comparisons provide contextual support for the projected trends but do not constitute formal external validation or calibration of the economic model. In particular, the model was not calibrated against independently observed future obesity-attributable healthcare expenditure or productivity losses. Consequently, the long-term economic estimates remain conditional on the selected forecasting specification and underlying epidemiological and economic assumptions.

### 2.4. Policy Scenarios

The model compared the projected future economic burden of obesity under alternative epidemiological scenarios to estimate the potential long-term economic benefits associated with sustained reductions in obesity prevalence. The primary base-case analysis compared two principal scenarios: (i) a future (status quo) scenario, in which obesity prevalence evolved according to projected epidemiological trends, and (ii) an annual reduction in obesity prevalence scenario, in which obesity prevalence declined annually according to predefined reduction targets. We evaluated both scenarios over a 30-year modelling horizon (2026–2055). To account for demographic change, the primary base-case analysis used a dynamically changing adult population based on annual population projections from the forecasting framework.

In the annual obesity-prevalence reduction scenarios, we examined annual relative reductions ranging from 1% to 20% to assess the potential economic consequences of alternative population-level obesity trajectories. A 5% annual relative reduction was selected as the primary illustrative scenario to provide a consistent reference point for the comparative economic analysis, while allowing both less and more ambitious reductions to be explored through scenario analyses. This assumption was not derived from a specific intervention, published effectiveness estimate or established national policy target. Rather, it represents a hypothetical scenario designed to demonstrate the potential economic implications of sustained reductions in population obesity prevalence. Importantly, maintaining a 5% annual relative reduction over the 30-year modelling horizon would result in a substantial cumulative decline in obesity prevalence; therefore, this scenario should not be interpreted as a forecast of achievable outcomes or as a realistic policy target.

Also, the 5% annual reduction refers to a relative reduction in obesity prevalence in each successive model year and should not be interpreted as a 5% reduction in body weight or BMI among individuals living with obesity. The model does not simulate individual weight trajectories, transitions between BMI categories or the clinical effectiveness of specific weight-management interventions. Accordingly, the scenarios were designed to examine the potential economic consequences of alternative population-level obesity-prevalence trajectories rather than to predict the outcomes of a particular intervention or policy programme.

The 5% annual relative reduction in adult obesity prevalence was specified as a hypothetical epidemiological scenario rather than a forecast of achievable population-level outcomes under existing obesity-management interventions. It aimed to quantify the potential economic consequences of a sustained reduction in obesity prevalence relative to the projected status quo. The substantial divergence between the two prevalence trajectories over the 30-year horizon therefore directly determines the resulting cumulative gross costs avoided. The model did not estimate the interventions, implementation requirements or expenditures necessary to achieve the assumed prevalence reduction.

For each projected year, we estimated total direct and indirect costs attributable to obesity separately under each scenario. The difference in total costs between the status quo and annual obesity prevalence reduction scenarios represented the annual economic benefit of reducing obesity prevalence. We then estimated cumulative undiscounted and discounted cost savings over the entire modelling horizon.

### 2.5. Obesity Management Interventions

#### 2.5.1. Obesity Pharmacotherapy

When first-line obesity management strategies, including dietary modification and physical activity, fail to achieve clinically meaningful weight reduction, clinicians may consider pharmacological treatment. Eligibility for reimbursed obesity pharmacotherapy in Greece is determined by criteria established by the Ministry of Health and the National Organisation for the Provision of Health Services (EOPYY). Currently, liraglutide 3 mg is the only anti-obesity medication reimbursed within the Greek public healthcare system. Reimbursement is restricted to adults with severe obesity (BMI ≥ 40 kg/m^2^) who also present with established cardiovascular disease or obstructive sleep apnoea requiring continuous positive airway pressure (CPAP) therapy [41]. Access to treatment requires prior authorisation through a formal approval process that involves submitting a named-patient request to a dedicated committee of the Supreme Health Council. This approval process must be renewed periodically to maintain eligibility for reimbursement [41].

We estimated the population meeting eligibility criteria for obesity pharmacotherapy from published literature and applied these estimates to the projected number of adults living with obesity for each year of the modelling horizon [42]. Despite the substantial number of individuals meeting clinical eligibility criteria, actual utilisation of reimbursed anti-obesity pharmacotherapy in Greece remains limited. In the absence of Greece-specific uptake data, we adopted a treatment uptake rate of 2.4% in the base-case analysis, based on published evidence [43].

#### 2.5.2. Bariatric Surgery

Bariatric surgery encompasses a range of surgical procedures intended to induce substantial and sustained weight loss through restriction of gastric volume and/or alteration of nutrient absorption [44]. The most common procedures are sleeve gastrectomy and gastric bypass, while adjustable gastric banding is performed less frequently [44].

According to national clinical guidance issued by the Central Health Council (KESY), eligibility for bariatric surgery in Greece includes adults with a BMI ≥ 40 kg/m^2^, as well as individuals with a BMI ≥ 35 kg/m^2^ and obesity-related comorbidities [45]. In routine clinical practice, bariatric surgery is generally considered a last-line therapeutic option following unsuccessful attempts at weight reduction through behavioural interventions and/or pharmacotherapy.

We estimated the population eligible for bariatric surgery using published evidence on the distribution of BMI categories and the prevalence of obesity-related comorbidities among adults living with obesity [42]. Despite the potentially large eligible population, bariatric surgery utilisation in Greece remains relatively limited. A recent systematic review and meta-analysis estimated that approximately 0.4% of eligible individuals undergo bariatric surgery annually, and the base-case analysis adopted this rate [46].

#### 2.5.3. Epidemiology of Obesity-Related Comorbidities

Obesity is not only a chronic disease but also a major risk factor for a broad spectrum of long-term health conditions [1,2,47]. Quantifying both the current burden attributable to obesity and the potential benefits of obesity reduction therefore requires comprehensive modelling of obesity-related complications (ORCs) and how changes in obesity prevalence translate into changes in disease burden over time. In the present study, we incorporated 36 major ORCs into the modelling framework. These included the 33 conditions examined in a previously published Greek obesity cost-of-illness study [11], plus three well-established obesity-related conditions: metabolic dysfunction-associated steatotic liver disease (MASLD), gout, and gallstones/cholelithiasis.

The included conditions spanned multiple clinical domains, including endocrine and metabolic disorders (e.g., type 2 diabetes, dyslipidaemia, polycystic ovary syndrome, MASLD and gout), cardiovascular diseases (e.g., angina, atrial fibrillation, heart failure, hypertension, ischaemic heart disease, myocardial infarction, pulmonary embolism and stroke), respiratory diseases (e.g., obstructive sleep apnoea and asthma), musculoskeletal disorders (e.g., osteoarthritis), renal disease (chronic kidney disease), mental health conditions (e.g., depression, anxiety and dementia), gastrointestinal disorders (e.g., gastro-oesophageal reflux disease and gallstones/cholelithiasis), as well as a broad range of obesity-associated malignancies. For cancer-related outcomes, prevalence estimates were obtained from the International Agency for Research on Cancer (IARC) Global Cancer Observatory (GLOBOCAN). Specifically, we used 5-year prevalence rather than annual incidence. This measure represents individuals diagnosed with the respective cancer in the preceding five years who were alive at the reference date and was selected to maintain consistency with the prevalence-based approach used for the other obesity-related comorbidities. Annual cancer incidence estimates reported separately by GLOBOCAN were not used in the model.

Compared with previous economic modelling studies evaluating obesity reduction and weight loss, which have generally focused on a narrower range of cardiometabolic and respiratory diseases, the present analysis adopted a substantially broader disease spectrum. Several previously published modelling studies from the United Kingdom, Spain, Sweden and Saudi Arabia have typically incorporated a core group of obesity-related conditions, including hypertension, dyslipidaemia, type 2 diabetes, cardiovascular disease, osteoarthritis, chronic kidney disease, atrial fibrillation, obstructive sleep apnoea and heart failure [13,15,16,18,19,20], while some analyses have focused almost exclusively on type 2 diabetes [17]. In contrast, the present study expanded the included disease spectrum to additionally capture metabolic, hepatic, renal, gastrointestinal, mental health and oncological outcomes. This broader scope enabled a more comprehensive estimate of the economic burden attributable to obesity and the potential cost savings from sustained reductions in adult obesity prevalence.

To quantify the proportion of disease burden attributable to obesity and the potential reduction under alternative annual reduction-in-obesity-prevalence scenarios, we applied the Population Attributable Fraction (PAF) methodology. The PAF estimates the proportion of cases of a specific condition that would theoretically not occur without obesity [28,29,30]. Its calculation requires three principal inputs: (1) obesity prevalence, (2) the relative risk associated with obesity for each comorbidity, and (3) the prevalence of each comorbidity within the population [28]. For each included condition, we calculated PAF values using standard epidemiological formulas. We then estimated the number of obesity-attributable cases by multiplying the PAF by the total number of individuals affected by each condition.

The following standard epidemiological formula was used to estimate the PAF for each obesity-related comorbidity:

PAF = [Po × (RR − 1)]/[1 + Po × (RR − 1)]

where

Po = prevalence of obesity;
RR = relative risk of developing comorbidity attributable to obesity.

The PAF framework enables comparison of the population-level obesity-attributable burden under alternative obesity-prevalence distributions. Because obesity prevalence is an explicit component of the PAF, the attributable fraction for each comorbidity was recalculated annually using the obesity prevalence corresponding to each modelled scenario. The resulting differences represent changes in the proportion of population-level comorbidity burden statistically attributable to obesity under alternative prevalence trajectories. These estimates should not be interpreted as indicating that existing comorbidities resolve contemporaneously when obesity prevalence declines. Instead, they quantify the model-estimated difference in obesity-attributable population burden under the specified counterfactual prevalence distribution. This interpretation aligns with the model’s aggregate, prevalence-based design and does not represent individual-level transitions between obesity, disease onset, remission, or subsequent disease states.

We identified prevalence estimates for each included comorbidity through a structured literature review using international scientific databases and, where available, national epidemiological sources. When multiple prevalence estimates were identified, lower estimates were preferentially selected to avoid systematically favouring higher-burden assumptions. However, this approach does not eliminate uncertainty or potential bias arising from differences in study populations, disease definitions, diagnostic criteria or measurement methods. The selected estimates were therefore treated as uncertain inputs rather than definitive lower bounds on disease prevalence. For comorbidities applicable to only one sex, we applied prevalence estimates to the corresponding sex-specific adult population rather than the total adult population. According to the Hellenic Statistical Authority, women accounted for 50.9% and men for 49.1% of the Greek population in 2025. Accordingly, the female and male adult populations used for sex-specific conditions were estimated by applying these proportions to the adult population included in the model. We derived relative risk estimates, representing the probability of developing each comorbidity among individuals living with obesity compared with individuals of normal weight [30], primarily from systematic reviews and meta-analyses or from studies conducted in countries with epidemiological profiles comparable to Greece, given the limited availability of Greece-specific relative risk data.

Finally, the number of individuals affected by each obesity-attributable comorbidity was estimated using the following formula:

PCOi = PCi × PAFi(1)

where

PCi = total population affected by the comorbidity;
PAFi = proportion of the comorbidity attributable to obesity.

The annual PAF is therefore an attribution measure rather than an individual-level disease-transition probability. Recalculating the PAF after a reduction in population obesity prevalence does not assume that an individual with an established chronic condition becomes disease-free after a change in BMI. Instead, it estimates the population-level burden attributable to obesity under the exposure distribution prevailing in each scenario.

By integrating obesity prevalence projections, comorbidity prevalence estimates, relative-risk parameters and PAF calculations, the model estimated the annual population-level obesity-attributable burden of each comorbidity under each modelled scenario. These estimates formed the basis for subsequent direct medical cost calculations and enabled comparison of the current and future economic burden associated with alternative obesity-prevalence trajectories.

### 2.6. Overview of Cost Categories

The present analysis incorporated direct and indirect cost components to provide a comprehensive assessment of the economic burden attributable to obesity and the potential economic benefits of sustained reductions in obesity prevalence. Adopting a societal perspective captured both healthcare system expenditures and productivity-related economic consequences associated with obesity.

Direct medical costs comprised three principal categories: (1) costs associated with obesity pharmacotherapy among eligible individuals receiving reimbursed treatment within the public healthcare system; (2) costs related to bariatric surgery, including procedural and perioperative healthcare expenditure; and (3) costs attributable to obesity-related comorbidities, estimated through application of population attributable fractions to disease-specific cost estimates.

To estimate the societal burden of obesity, the analysis also incorporated indirect costs arising from absenteeism and presenteeism. As both outcomes relate specifically to workplace productivity, these costs were restricted to the estimated employed population living with obesity rather than being applied to all adults living with obesity. Absenteeism reflected productivity losses from work absence due to obesity-related morbidity, whereas presenteeism captured reduced productivity while remaining at work despite impaired health.

Collectively, these cost components enabled estimation of the total annual economic burden attributable to obesity in Greece and the corresponding economic benefits achievable under alternative obesity-reduction scenarios.

### 2.7. Direct Medical Costs

The analysis incorporated direct medical costs for both obesity management and treatment of obesity-related comorbidities. Direct costs comprised three principal components: (1) pharmacotherapy costs for eligible individuals receiving reimbursed anti-obesity medication; (2) bariatric surgery costs; and (3) healthcare costs associated with the management of obesity-related comorbidities. This approach enabled a comprehensive estimation of healthcare system expenditure attributable to obesity.

We estimated annual pharmacotherapy cost per treated patient using the retail price of liraglutide 3 mg reported in the official national price bulletin valid for 2025. To estimate payer-perspective cost, we deducted the statutory patient co-payment rate (25%) from the retail price. We did not incorporate confidential rebates and discounts because this information is not publicly available and cannot be reliably estimated. We then applied the resulting gross reimbursement cost per treated patient to the projected number of individuals receiving obesity pharmacotherapy in each year of the model.

We derived the cost of bariatric surgery from the Greek Diagnosis-Related Group (DRG) reimbursement system. Specifically, we applied the average reimbursement value of DRG codes corresponding to major bariatric procedures, with and without complications. These reimbursement tariffs capture payer-borne expenditure, including acute hospitalisation, surgical intervention, postoperative care, and rehabilitation services, providing a comprehensive estimate of the cost per bariatric procedure. However, it should be noted that these tariffs were used as proxies for the payer-borne costs of the indexed surgical hospitalisation. We did not separately model additional expenditure outside the reimbursed hospital episode, including subsequent outpatient follow-up and later complications.

To estimate the direct medical costs associated with obesity-related comorbidities, we conducted a structured literature review to identify annual per-patient cost estimates for each included condition. Direct medical costs included medication, diagnostic monitoring, laboratory testing, outpatient visits, inpatient care, emergency department utilisation, and hospitalisations directly related to managing each comorbidity. When multiple annual cost estimates were available for a given comorbidity, we preferentially selected lower estimates to avoid systematically favouring higher-cost assumptions. However, this selection strategy does not eliminate uncertainty or potential bias arising from differences in study populations, costing methodologies, healthcare settings or the scope of included resources. Also, in the absence of a Greece-specific estimate for the annual direct medical cost of clinically diagnosed anxiety, the annual cost was conservatively assumed to be 50% of that for depression. This approach was previously adopted in a cost-of-illness study of obesity in Greece and was retained to ensure methodological consistency while avoiding cost overestimation [11]. For MASLD, we also revised the annual healthcare-cost input after assessing the correspondence between the modelled epidemiological population and the population represented by the original cost source. The revised input was obtained from a French observational cost study based on the CONSTANCES cohort, linked to the national healthcare claims database. The analysis included 14,437 adults with MASLD, identified using the fatty liver index, including individuals with MASH. Mean annual healthcare expenditure was EUR 1860 per individual (SD, EUR 4634). In the absence of a comparable Greece-specific MASLD healthcare-cost estimate, we used French evidence as a proxy and adjusted it to 2025 price levels using the applicable inflation index. This broader population-based estimate better matches the modelled MASLD population than the previously used NASH-specific treatment cost. However, the estimate represents total healthcare expenditure among individuals with MASLD, including expenditure associated with coexisting conditions, rather than the incremental cost attributable exclusively to MASLD. Consequently, potential overlap with other obesity-related comorbidity costs was recognised as a source of uncertainty in the aggregate cost estimates

For cancer-related outcomes, the economic analysis was aligned with the prevalence-based epidemiological approach. We applied the annual cancer cost parameter to the corresponding obesity-attributable 5-year prevalent cancer population rather than to annual incident cases. The resulting estimate therefore represents the annual healthcare burden associated with prevalent cancer cases included in the model.

We estimated the total direct cost attributable to obesity for each comorbidity by multiplying the number of obesity-attributable cases, derived from the PAF analysis, by the annual direct medical cost per patient for that condition. Costs were subsequently aggregated across all included comorbidities to estimate the total annual comorbidity-related expenditure attributable to obesity using the following formula:

TCi = PCOi × DCi(2)

where

TCi = total direct cost attributable to obesity for comorbidity i;
PCOi = number of individuals affected by comorbidity i attributable to obesity;
DCi = annual direct medical cost per patient for comorbidity i.

The resulting estimates represent annual population-level healthcare expenditure statistically attributable to obesity under each modelled prevalence trajectory. They should therefore be interpreted as comparative estimates of attributable economic burden rather than as patient-level predictions that healthcare expenditure ceases immediately following a reduction in BMI.

Where necessary, we inflated cost estimates from earlier years to 2025 price levels using the Consumer Price Index (CPI) published by the Hellenic Statistical Authority (ELSTAT) to ensure methodological consistency and comparability across cost inputs [31].

We excluded certain cost categories from the analysis for methodological reasons or due to limited data availability. We did not include routine primary care and specialist consultation costs separately, assuming that healthcare utilisation patterns after diagnosis of a specific comorbidity would not differ substantially between individuals with and without obesity. The base-case analysis excluded costs associated with obesity pharmacotherapies currently financed through the Recovery and Resilience Facility (RRF), including tirzepatide and semaglutide, because this funding mechanism is temporary and falls outside the routine public healthcare budget. In addition, we excluded direct non-medical costs, including transportation expenses, gym subscriptions, and specialised equipment, because robust Greece-specific evidence was lacking and the base-case analysis focused on healthcare system expenditure.

### 2.8. Indirect Costs: Productivity Losses Due to Absenteeism and Presenteeism

Indirect costs attributable to obesity were estimated to capture productivity losses arising from impaired work participation and reduced occupational performance. Specifically, the analysis incorporated productivity losses from absenteeism (workdays lost due to illness) and presenteeism (reduced productivity while at work). Because Greece lacks data that directly quantify obesity-attributable productivity losses, the methodological approach aligned with a recent obesity cost-of-illness study conducted in Greece [11].

Productivity loss estimates were primarily derived from Gupta et al., which applied the human capital approach to estimate absenteeism and presenteeism costs across BMI categories using data from five major European economies [48]. In that study, individuals were stratified according to BMI class, and the incremental productivity losses associated with obesity relative to normal-weight individuals were estimated in monetary terms [48].

In the present analysis, adults living with obesity were categorised according to standard BMI classes: class I (30–34.9 kg/m^2^), class II (35–39.9 kg/m^2^), and class III (≥40 kg/m^2^). For each BMI class, the corresponding incremental annual productivity cost relative to normal-weight individuals was applied. Because absenteeism and presenteeism represent workplace productivity losses, these costs were restricted to the estimated employed population living with obesity. To ensure consistency between the population denominator and the employment-rate definition, the estimation of obesity-attributable productivity losses was restricted to the working-age population aged 20–64 years. Specifically, the total number of adults living with obesity was adjusted using the proportion of the adult population aged 20–64 years, estimated at 69.17% in 2025. The resulting working-age population living with obesity was subsequently multiplied by the national employment rate of 71.0% for individuals aged 20–64 years. Because obesity-prevalence estimates specific to this age group were unavailable, we assumed overall adult obesity prevalence was representative of the working-age population. These shares were applied consistently across projected years with this assumption being explicitly acknowledged as a source of uncertainty and a limitation.

We then distributed the estimated employed population living with obesity across obesity classes I, II, and III using the corresponding BMI-class proportions of 56.32%, 26.81%, and 16.86%, respectively. Class-specific incremental absenteeism and presenteeism costs were then applied to estimate total obesity-attributable productivity losses. Incremental costs were calculated relative to individuals with normal BMI and adjusted to reflect the Greek economic context and the 2025 cost year.

The working-age population adjustment was incorporated consistently into both the status quo and hypothetical obesity-prevalence reduction scenarios throughout the modelling horizon, ensuring that productivity-related economic outcomes were estimated using a population denominator aligned with the employment-rate definition.

Also, as the original productivity estimates were derived from higher-income European settings, we adjusted them to ensure applicability to the Greek economic context. Specifically, we scaled cost estimates using the ratio of Greece’s gross domestic product (GDP) per capita to the GDP per capita of the reference countries in the base year of the original study, aligning productivity valuations with national economic conditions [49]. We then inflated all adjusted productivity cost estimates to 2025 price levels using the Consumer Price Index (CPI) published by the Hellenic Statistical Authority (ELSTAT) [31].

We held the proportions of adults living with obesity across BMI classes I, II, and III constant throughout the projection horizon, as longitudinal Greece-specific evidence was insufficient to estimate changes in the distribution of obesity severity under alternative prevalence trajectories. Similarly, BMI-class-specific annual absenteeism and presenteeism costs were maintained at their 2025 values in real terms, and the employment-rate assumption was held constant. These assumptions enabled consistent comparisons across scenarios but did not capture potential changes in obesity severity, employment participation, wages, workplace conditions or productivity over time. Consequently, the estimated productivity losses remain conditional on these parameters remaining stable.

We estimated total indirect costs by multiplying the adjusted annual per-person productivity loss for each BMI class by the number of working individuals in each obesity category included in the model. This approach allowed us to estimate the aggregate societal productivity burden attributable to obesity under both the status quo and alternative annual reduction-in-obesity-prevalence scenarios.

Costs associated with premature mortality were not incorporated into the analysis. Reliable Greece-specific data regarding deaths directly attributable to obesity, as well as the average age at death among individuals living with obesity, are currently unavailable, limiting the ability to estimate years of life lost and associated productivity losses with sufficient methodological accuracy. Furthermore, most previously published economic modelling studies evaluating the economic benefits of weight reduction have similarly excluded premature mortality from indirect cost calculations, focusing predominantly on morbidity-related productivity losses [15,16,17,18,19,20,21,22]. Consequently, exclusion of premature mortality in the present analysis reflects both data limitations and consistency with existing modelling practice. Although this approach may underestimate the total societal burden attributable to obesity, it avoids introducing substantial uncertainty arising from speculative assumptions and limited empirical evidence.

### 2.9. Primary Economic Outputs

The model generated annual and cumulative economic outcomes for each examined scenario over the 2026–2055 modelling horizon. The primary economic outcomes included total annual direct and indirect costs attributable to obesity, cumulative undiscounted costs across the entire study period, and cumulative discounted costs applying an annual discount rate. The difference in total obesity-attributable costs between the future (status quo) scenario and each alternative obesity-prevalence trajectory was interpreted as the model-estimated potential economic saving associated with the lower population prevalence of obesity under the assumptions of the prevalence-based PAF framework. These differences were subsequently aggregated to estimate cumulative undiscounted and discounted potential cost savings over the modelling horizon. They should not be interpreted as directly observed savings or as implying contemporaneous remission of established obesity-related disease. Also, the differences in projected economic burden between the status quo and alternative obesity-prevalence scenarios were interpreted as potential gross costs avoided under the specified hypothetical prevalence trajectories. Although the model incorporated projected expenditure associated with obesity pharmacotherapy and bariatric surgery, it did not estimate the full implementation, delivery or maintenance costs of the interventions and policies that would be required to achieve the assumed population-level reductions in obesity prevalence. Consequently, the estimated economic benefits do not represent net savings, cost-effectiveness estimates or returns on investment.

In addition, the model estimated the annual economic cost per individual living with obesity under each scenario throughout the simulation period. This metric enabled assessment of how projected changes in obesity prevalence, disease burden and intervention uptake influenced the average economic burden per affected individual over time.

### 2.10. Methodological Framework for Sensitivity Analyses

As the baseline estimates of the economic model were derived from multiple epidemiological, clinical and economic sources, the model outputs were inherently subject to parameter and structural uncertainty. To assess the robustness of the base-case findings, we conducted deterministic and probabilistic sensitivity analyses.

Scenario analyses examined how the magnitude of annual obesity-prevalence reduction, analytical perspective, time horizon, and discount rate affect estimated economic outcomes. To explore the temporal dynamics of economic benefits, we conducted additional analyses across alternative modelling horizons of 5, 10, 15, 20 and 25 years. We selected these shorter horizons to align more closely with government budget cycles and policy decision-making frameworks, facilitating interpretation of the potential economic benefits associated with obesity prevention and management interventions.

An additional scenario analysis was conducted to assess the implications of using crude rather than age-standardised adult obesity prevalence. We obtained annual crude prevalence estimates for Greece from the WHO Global Health Observatory and independently extrapolated them over 2025–2055 using the same exponential smoothing (ETS) forecasting framework used in the primary analysis. We applied the resulting annual crude prevalence projections to the corresponding projected adult population to estimate the number of individuals living with obesity. We then recalculated all prevalence-dependent calculations, including obesity-attributable comorbidity burden, population-attributable fractions, direct medical costs, and indirect productivity losses, under both the status quo and hypothetical prevalence-reduction trajectories. We held constant the annual relative prevalence-reduction assumptions, demographic projections, disease-specific relative risks, unit costs, discount rate, and remaining model parameters across the two specifications. This approach isolated the prevalence measure’s effect on the estimated economic burden and cumulative gross costs avoided while preserving comparability with the primary analysis. The crude-prevalence scenario was analysed independently and was not incorporated into the probabilistic sensitivity analysis.

Because discounting assumptions may substantially influence long-term economic projections, additional scenario analyses examined the net present value (NPV) of projected cost savings under alternative annual discount rates ranging from 1% to 30%. These analyses assessed the stability of the economic conclusions under varying assumptions about time preference and the future valuation of healthcare expenditure.

We then performed a deterministic sensitivity analysis (DSA) to evaluate how uncertainty in individual parameters affects model outcomes. All key input parameters (*n* = 132) were varied independently by ±20% relative to their base-case values. We estimated the impact on cumulative discounted cost savings and summarised the findings using a tornado diagram to identify the parameters exerting the greatest influence on model outputs. We then reported the ten most influential parameters.

We excluded discount rates from the one-way deterministic sensitivity analysis because we assessed their influence separately through dedicated discount-rate scenarios. Uncertainty in projected obesity prevalence was explored using alternative forecasting bounds and prevalence-reduction trajectories rather than through a single-parameter variation in the tornado analysis. However, the alternative prevalence-reduction scenarios primarily assess uncertainty in the assumed policy trajectory and do not fully substitute for independent variation in baseline obesity prevalence or uncertainty in the underlying status quo forecast. The results of the deterministic analysis should therefore be interpreted alongside the dedicated scenario analyses rather than as a comprehensive ranking of all sources of model uncertainty.

Finally, we conducted a probabilistic sensitivity analysis (PSA) to account for joint parameter uncertainty and evaluate the model’s robustness to simultaneous variation in key inputs. We performed a second-order Monte Carlo simulation with 1000 iterations. We assigned probability distributions in line with established health-economic modelling practice: comorbidity prevalence parameters were modelled using beta distributions, cost parameters using gamma distributions, and relative risk estimates using log-normal distributions [50]. We modelled uptake rates for obesity pharmacotherapy and bariatric surgery using uniform distributions bounded between 0.5% and 5%. We modelled adult population and obesity prevalence estimates using a uniform distribution with lower and upper bounds reflecting the simulated lower and upper confidence intervals. We assumed a base-case standard error of 10%; however, we also analysed whether changes in standard error (5–30%) meaningfully affected the PSA results.

We generated PSA mean estimates and corresponding 95% confidence intervals for all principal economic outcomes, including total costs, cumulative undiscounted cost savings, and cumulative discounted cost savings. We estimated confidence intervals using the percentile method.

The uncertainty analyses distinguished between parameter uncertainty and uncertainty arising from the modelling framework’s structural assumptions. Deterministic and probabilistic sensitivity analyses examined variation in selected epidemiological and economic inputs within the specified model structure, whereas scenario analyses explored alternative modelling assumptions, including the use of crude rather than age-standardised obesity prevalence. The probabilistic sensitivity analysis did not fully account for uncertainty in disease latency, multimorbidity, correlations among epidemiological and cost parameters, or potential changes in underlying relationships over the 30-year projection horizon. Accordingly, the reported 95% uncertainty intervals reflect uncertainty conditional on the selected model specification and parameter distributions, not the full range of plausible economic outcomes.

## 3. Results

### 3.1. Baseline Demography, Epidemiology and Economic Burden of Adult Obesity in 2025

In 2025, Greece’s total population was estimated at 10,372,335 individuals, of whom 83.96% (8,708,555) were adults [36]. The estimated prevalence of adult obesity was 29.24%, corresponding to 2,546,667 adults living with obesity.

#### 3.1.1. Obesity Management

Among adults living with obesity, 16.86% (429,492 individuals) were classified as having class III obesity (BMI ≥ 40 kg/m^2^) [42]. Of these individuals, 5.90% were estimated to have established cardiovascular disease [51], while 26.20% were estimated to have obstructive sleep apnoea requiring continuous positive airway pressure (CPAP) therapy [52,53]. Based on current national reimbursement criteria, an estimated 137,867 individuals were eligible for reimbursed obesity pharmacotherapy. Nevertheless, treatment uptake remained limited, with only 2.40% of eligible individuals receiving pharmacotherapy, corresponding to 3309 treated patients in 2025 [43,54].

In 2025, the retail price of liraglutide 3 mg was EUR 217.31 per pack [55]. After deducting the statutory 25% patient co-payment, the payer cost was estimated at EUR 162.98 per pack. To reflect real-world treatment persistence, we applied an adjustment based on evidence indicating a mean treatment persistence of 6.3 months [56]. Consequently, we assumed an average annual utilisation of six packs per treated patient, resulting in an annual pharmacotherapy cost of EUR 977.90 per patient and a total obesity pharmacotherapy expenditure of EUR 3.23 million in 2025. This estimate assumes no further pharmacotherapy costs after treatment discontinuation and does not account for costs of treatment reinitiation, switching to alternative anti-obesity medications, or subsequent treatment episodes. Consequently, the estimated pharmacotherapy expenditure is likely to be conservative.

Regarding bariatric surgery, 26.81% of adults living with obesity were classified as BMI class II (BMI 35–39.9 kg/m^2^), of whom approximately 25.70% were estimated to present with at least one obesity-related comorbidity [42,57]. Combined with individuals with BMI ≥ 40 kg/m^2^, an estimated 604,967 adults were eligible for bariatric surgery. Despite this sizeable eligible population, utilisation remained low, with only 0.40% undergoing surgery annually, corresponding to 2420 procedures in 2025 [58]. Using the mean reimbursement value of the relevant DRG tariffs for procedures with and without complications, the average cost per bariatric intervention was estimated at EUR 2383 [11], yielding a total annual expenditure for bariatric surgery of EUR 5.76 million.

#### 3.1.2. Obesity-Related Comorbidities

Across the 36 obesity-related comorbidities included in the model, the most prevalent conditions in the adult population were hypertension (1,541,414 cases), dyslipidaemia (1,454,329 cases), MASLD (848,205 cases), asthma (792,479 cases), type 2 diabetes (644,433 cases), and chronic kidney disease (627,887 cases). The highest PAFs were observed for obstructive sleep apnoea (64.38%), type 2 diabetes (51.01%), Osteoarthritis (50.94%), MASLD (42.52%), dyslipidaemia (35.23%) and hypertension (25.49%).

After applying the PAF methodology, the largest numbers of obesity-attributable cases were observed for dyslipidaemia (512,360 cases), hypertension (392,945 cases), MASLD (360,690 cases), type 2 diabetes (328,699 cases), and asthma (168,007 cases). From an economic perspective, type 2 diabetes, dyslipidaemia, MASLD, hypertension, ischaemic heart disease and angina collectively accounted for approximately 68.86% of total direct comorbidity-related expenditure attributable to obesity. Overall, total direct medical costs attributable to obesity-related comorbidities were estimated at EUR 3.682 billion in 2025. Table 1 and Table 2 summarise key epidemiology and cost inputs.

#### 3.1.3. Indirect Costs

Among the 2,546,667 adults living with obesity, 56.32% were classified as obesity class I (BMI 30–34.9 kg/m^2^), 26.81% as obesity class II (BMI 35–39.9 kg/m^2^), and 16.86% as obesity class III (BMI ≥ 40 kg/m^2^) [42]. After adjusting productivity-loss estimates from Gupta et al. [48] to Greek GDP per capita and inflating them to 2025 price levels, the annual absenteeism cost per individual was estimated at EUR 77.50 for obesity class I, EUR 249.15 for obesity class II, and EUR 514.95 for obesity class III. After adjusting for the working-age population, the estimated number of employed adults living with obesity in 2025 was approximately 1.25 million, distributed across obesity classes I, II, and III. The corresponding annual obesity-attributable absenteeism cost was estimated at EUR 246.75 million. Similarly, adjusted presenteeism costs per individual were estimated at EUR 74.30 for obesity class I, EUR 223.53 for obesity class II and EUR 780.76 for obesity class III, resulting in total presenteeism-related productivity losses of EUR 291.97 million. Overall, total indirect costs attributable to obesity were estimated at EUR 538.72 million in 2025.

#### 3.1.4. Total Economic Burden

Overall, the total societal economic burden attributable to adult obesity in Greece in 2025 was estimated at EUR 4.229 billion, of which EUR 3.691 billion (87.26%) represented direct medical costs and EUR 538.72 million (12.74%) reflected productivity losses. The average annual economic burden per capita was estimated at EUR 407.80, whereas the corresponding figure for adults living with obesity was EUR 1660.93. Relative to national economic output [130], obesity-related costs corresponded to approximately 1.70% of Greece’s gross domestic product (GDP) in 2025.

Table 3 presents the total economic burden estimates.

### 3.2. Projected Adult Obesity Prevalence

**Figure 2:**
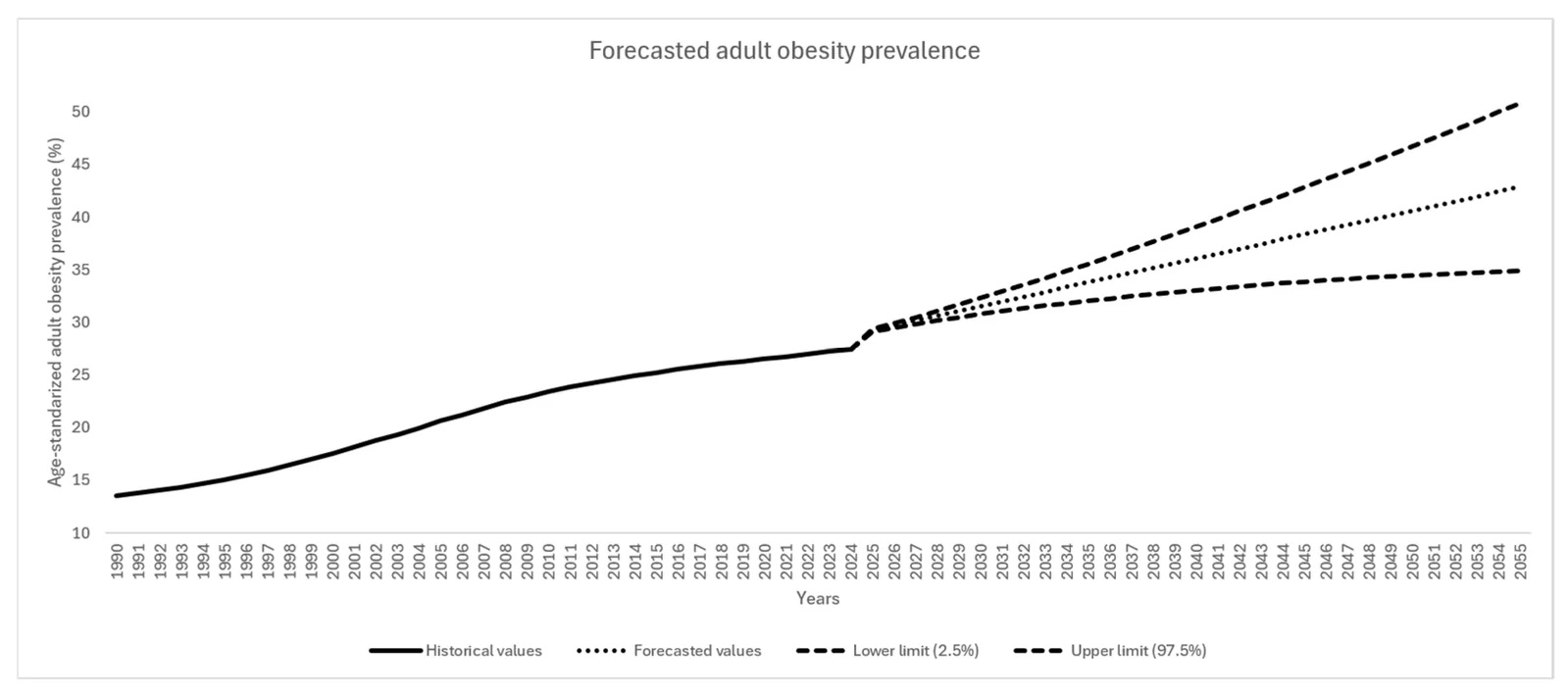
Historical and projected adult obesity prevalence in Greece, 1990–2055. The lower (2.5th percentile) and upper (97.5th percentile) limits represent the bounds of the 95% forecast interval, reflecting uncertainty around the projected adult obesity prevalence estimates.

Projected adult obesity prevalence in Greece showed a sustained, progressively increasing trajectory throughout the study horizon. Starting from the last observed prevalence estimate of 27.43% in 2024, adult obesity prevalence was projected to increase to 29.24% by 2025, 31.51% by 2030, 36.05% by 2040 and 42.87% by 2055 (Figure 2). Overall, this corresponds to an absolute increase of 13.62 percentage points and a relative increase of 46.58% between 2025 and 2055, indicating a substantial worsening of the obesity epidemic under the status quo scenario. These projections align with historical epidemiological trends in Greece, where adult obesity prevalence increased from 17.54% in 2000 to 27.43% in 2024, a relative increase of 56.39% over the examined period.

The projected increase followed an approximately linear trajectory, with a relatively stable annual increase of approximately 0.45 percentage points. This closely mirrors historical patterns, as the mean annual increase in adult obesity prevalence between 1990 and 2024 was approximately 0.41 percentage points. Importantly, we observed no evidence of a prevalence plateau over the projection horizon, suggesting that obesity prevalence in Greece is likely to continue increasing without effective population-level public health strategies.

Forecast uncertainty increased progressively over time, as reflected in the widening confidence intervals around the projected estimates. The confidence interval widened from 0.31 percentage points in 2025, then to 15.92 percentage points by 2055, reflecting the increasing uncertainty inherent in long-term epidemiological forecasting. By 2040, the lower-bound prevalence estimate was 33.03%, while the upper-bound estimate was 39.07%. Similarly, by 2055, projected obesity prevalence ranged from 34.91% to 50.83%. Although projected prevalence increased across the examined uncertainty bounds, the widening prediction intervals highlight substantial uncertainty about the magnitude of obesity prevalence several decades into the future. Accordingly, the long-term estimates should be interpreted as conditional projections based on historical trends rather than precise forecasts of future prevalence.

The obesity prevalence estimates in this study broadly align with projections in the Global Burden of Disease (GBD) 2021 forecasting study [131]. Specifically, the GBD analysis estimated that the age-standardised prevalence of overweight and obesity among adults aged ≥25 years in Greece would increase from 49.75% in 1990 to 78.20% by 2050, corresponding to an overall increase of 58.03% [131]. Focusing on obesity, the GBD study projected adult obesity prevalence to rise from 12.44% in 1990 to 34.41% in 2050, a relative increase of 178.14%. Importantly, the GBD estimate for 2050 falls within the confidence interval generated by the present analysis for the same year (34.45–46.74%), thereby supporting the external validity of the projected estimates. Furthermore, the GBD study estimated an absolute increase of 10.22 percentage points in adult obesity prevalence between 2021 and 2050, corresponding to an approximate annual increase of 0.35 percentage points [131]. This closely aligns with the 0.45 percentage-point annual increase estimated in the present study.

### 3.3. Impact of Different Annual Reduction in Obesity Prevalence Thresholds on Obesity Prevalence

Across all scenarios, adult obesity prevalence showed substantial, sustained reductions relative to the projected status quo trajectory, with the magnitude of decline increasing proportionally with the assumed annual obesity-reduction rate.

Under the base-case 5% annual obesity-reduction scenario, adult obesity prevalence was projected to decline from 29.24% in 2025 to 22.63% by 2030, 13.55% by 2040 and 6.28% by 2055. Over the full modelling horizon, mean obesity prevalence was estimated at 15.02%, an absolute reduction of 22.97 percentage points and a cumulative decrease rate of 78.54%. This translated to an average annual reduction of 0.78 percentage points. The year-by-year calculation of gross costs avoided and their corresponding discounted values is presented in Supplementary Table S5.

Lower-intensity annual reduction scenarios produced proportionally smaller but still meaningful reductions in obesity prevalence. Under the 1% and 2% annual reduction scenarios, the mean projected obesity prevalence was estimated at 25.25% and 21.95%, corresponding to cumulative reductions of 26.03% and 45.45%, respectively. In contrast, more intensive intervention scenarios generated substantially larger epidemiological effects. Under the 10% annual reduction scenario, mean obesity prevalence declined to 9.07%, while under the 15% and 20% scenarios, it further decreased to 6.25% and 4.71%, respectively, corresponding to cumulative reductions exceeding 95%.

### 3.4. Economic Burden of Adult Obesity and Potential Cost-Savings Under Varying Annual Reduction in Obesity Prevalence Scenarios

Building on the projected epidemiological burden of obesity and the estimated reductions under alternative scenarios, the following section quantifies the economic consequences. Using the population-level PAF framework described above, the analysis compares obesity-attributable direct healthcare expenditure and indirect productivity losses under alternative obesity-prevalence trajectories. The analysis reports differences between scenarios as model-estimated potential economic savings and should be interpreted within the assumptions of the prevalence-based framework.

#### 3.4.1. Economic Burden of Obesity Under the Future Status Quo Scenario

Under the future status quo scenario, and accounting for a dynamically shifting and declining adult population, the total economic burden attributable to obesity in Greece is projected to increase steadily throughout the study horizon. Total annual obesity-related costs are projected to rise from EUR 4.26 billion in 2026 to EUR 5.11 billion by 2055, an increase of approximately EUR 846.28 million and EUR 28.22 million per year (Supplementary Figure S1). Across the full modelling horizon, the cumulative economic burden is estimated at EUR 141.64 billion, with a mean annual cost of EUR 4.72 billion and a median of EUR 4.74 billion.

Direct medical costs account for the largest share of the overall economic burden, representing about 86.38% of total obesity-related expenditure. These costs are projected to rise from EUR 3.72 billion in 2026 to EUR 4.38 billion in 2055, an absolute increase of EUR 657.56 million. Nearly all direct healthcare expenditure is attributable to obesity-related comorbidities (ORCs), which account for approximately 86.16% of total cumulative expenditure, elucidating the central role of chronic obesity-related diseases in driving healthcare costs. Regarding the comorbidities contributing most to ORC-related expenditure, a relatively small number of high-burden chronic conditions—primarily type 2 diabetes, angina, hypertension, ischaemic heart disease, dyslipidaemia, and MASLD—collectively account for 68.86% of total ORC costs over the projection period.

By contrast, obesity-specific interventions remain economically marginal within the current healthcare landscape. Pharmacotherapy and bariatric surgery collectively contribute less than 0.21% of total projected expenditure.

Indirect costs are a growing component of the total economic burden, accounting for approximately 13.62% of forecasted cumulative obesity-related costs. Over the study horizon, cumulative productivity losses are projected to reach approximately EUR 19.29 billion. Annual indirect costs are projected to increase from EUR 545.81 million in 2026 to EUR 734.74 million in 2055, an absolute increase of EUR 188.93 million and an annual increase of EUR 6.30 million. Productivity losses are driven by presenteeism, which accounts for approximately 7.38% of total cumulative costs, compared with 6.24% attributable to absenteeism, highlighting the substantial economic consequences of impaired workplace productivity among working individuals living with obesity.

#### 3.4.2. Economic Burden Under the 5% Annual Obesity Prevalence Reduction Scenario

Under the base-case scenario, assuming a sustained 5% annual reduction in adult obesity prevalence, the economic burden attributable to obesity was projected to decline substantially and continuously throughout the study horizon. Accounting for a dynamically shifting and declining adult population, total annual obesity-related costs were projected to fall from EUR 4.06 billion in 2026 to EUR 1.12 billion by 2055, an absolute reduction of EUR 2.94 billion and an absolute annual decrease of approximately EUR 98.07 million (Supplementary Figure S2).

Across the full modelling horizon, cumulative obesity-related expenditure was estimated at EUR 70.52 billion, representing almost half of the cumulative burden projected under the status quo scenario.

Direct medical costs remained the dominant contributor to the total economic burden, accounting for approximately 88.91% of total cumulative expenditure under the intervention scenario. These costs were projected to decrease from EUR 3.55 billion in 2026 to EUR 1.02 billion by 2055, an absolute reduction of EUR 2.53 billion and an annual decrease of EUR 84.41 million. As in the status quo scenario, obesity-related comorbidities (ORCs) continued to account for most direct healthcare expenditure and at approximately 88.72% of cumulative total costs throughout the study period. A relatively limited number of high-burden chronic conditions—including type 2 diabetes, angina, hypertension, ischaemic heart disease, dyslipidaemia, and MASLD—collectively accounted for nearly 80% of total ORC-related cost reductions.

By contrast, obesity-specific interventions remained economically marginal. Pharmacotherapy and bariatric surgery collectively accounted for less than 0.1% of total cumulative expenditure, despite exhibiting larger proportional reductions over time, primarily reflecting their limited baseline contribution to overall healthcare costs.

Indirect costs declined even more steeply than direct medical costs. Annual productivity losses were projected to decrease from EUR 510.59 million in 2026 to EUR 107.59 million by 2055, an absolute reduction of EUR 403.01 million and an annual decrease of EUR 13.43 million. Across the entire study horizon, cumulative indirect costs were estimated at EUR 7.82 billion, accounting for approximately 11.09% of the total cumulative economic burden. Productivity losses were driven by presenteeism, which accounted for EUR 4.24 billion cumulatively, followed by absenteeism (EUR 3.58 billion), indicating that impaired workplace productivity remains a major societal consequence of obesity even with substantial reductions in obesity prevalence.

#### 3.4.3. Incremental Economic Impact of the 5% Annual Obesity Reduction Compared with the Status Quo Scenario

The comparison between the base-case 5% annual obesity-reduction scenario and the projected status quo trajectory demonstrated progressively increasing economic savings over time, reflecting the cumulative impact of sustained reductions in obesity prevalence and ORCs. The divergence between scenarios widened consistently throughout the modelling horizon, indicating that the economic benefits of obesity reduction intensified over time due to compounding epidemiological effects.

At the aggregate level, total annual obesity-related expenditure was projected to be EUR 198.68 million lower in 2026, increasing to EUR 941.52 million by 2030, EUR 2.46 billion by 2040, and EUR 3.99 billion by 2055 relative to the status quo scenario. Across the study period, cumulative undiscounted cost savings were estimated at EUR 71.12 billion, corresponding to a mean annual reduction of EUR 2.37 billion and a median annual reduction of EUR 2.46 billion (Supplementary Figure S3).

Direct medical costs accounted for the largest proportion of avoided expenditure, representing approximately 83.87% of total cumulative savings. Total cumulative direct-cost savings were estimated at EUR 59.65 billion, with most attributable to reductions in obesity-related comorbidities. By 2055, annual direct-cost savings were projected to reach EUR 3.35 billion, an absolute annual increase of about EUR 106.22 million. Most of the avoided healthcare expenditure was concentrated in a relatively small number of high-burden cardiometabolic and metabolic conditions. Specifically, type 2 diabetes, angina, hypertension, ischaemic heart disease, dyslipidaemia, and MASLD collectively accounted for nearly 80% of total ORC-related savings, highlighting their leading contribution to obesity-attributable healthcare expenditure.

Indirect-cost savings were also substantial, with cumulative avoided productivity losses estimated at EUR 11.47 billion, accounting for approximately 16.13% of total cumulative savings. Annual indirect-cost savings increased from EUR 35.22 million in 2026 to approximately EUR 627.15 million by 2055. Productivity-related benefits were driven by reductions in presenteeism (EUR 6.21 billion cumulatively), followed by absenteeism (EUR 5.25 billion cumulatively), reflecting the substantial macroeconomic benefits of sustained obesity reduction and improved workforce productivity.

From an economic perspective, applying a 5% annual discount rate yielded an NPV of approximately EUR 29.25 billion in avoided costs over the study horizon. Discounted annual savings increased progressively, peaking around 2048 at approximately EUR 1.13 billion annually before gradually declining in present-value terms due to the cumulative effect of discounting.

From a macroeconomic perspective, annual undiscounted cost savings from the 5% annual obesity-reduction scenario were projected to increase over time, reaching about 1.18% of projected national gross domestic product (GDP) by 2055. The mean savings per obese individual over the study’s time horizon (2026–2055) was estimated at EUR 783.49.

Under the base-case 5% annual obesity-reduction scenario, obesity prevalence declined from 29.24% in 2025 to 6.28% by 2055, corresponding to an absolute reduction of 22.96 percentage points. With cumulative undiscounted cost savings of EUR 71.12 billion, this translates to about EUR 3.11 billion in savings per one percentage-point reduction in adult obesity prevalence. After applying the 5% annual discount factor, the discounted savings amount to approximately EUR 1.28 billion in NPV per one percentage-point reduction in obesity prevalence.

Even under the most conservative 1% annual obesity-reduction scenario, obesity prevalence declined from 29.24% to 21.63% between 2025 and 2055, corresponding to an absolute reduction of 7.58 percentage points. This generated approximately EUR 32.04 billion in cumulative undiscounted cost savings, equivalent to EUR 4.23 billion per one-percentage-point reduction in obesity prevalence, while discounted savings reached EUR 12.87 billion, corresponding to approximately EUR 1.70 billion in NPV savings per one-percentage-point reduction.

### 3.5. Sensitivity Analyses

#### 3.5.1. Scenario Analyses

*Cost-savings under varying annual reduction in obesity prevalence thresholds*

Projected economic savings increased monotonically with the magnitude of the assumed annual reduction in obesity prevalence, demonstrating a strong and consistent relationship between sustained reductions in adult obesity prevalence and long-term economic benefit. Under the shifting-population scenario, cumulative undiscounted cost-savings ranged from EUR 33.04 billion under the 1% annual obesity-reduction scenario to EUR 121.22 billion under the 20% scenario. Correspondingly, discounted savings increased from EUR 12.87 billion to EUR 54.93 billion

More ambitious obesity-reduction scenarios generated progressively larger potential gross savings. Compared with the base-case 5% scenario, the 10%, 15% and 20% annual reduction scenarios increased cumulative discounted savings to EUR 42.26 billion, EUR 50.01 billion and EUR 54.93 billion, respectively. Nevertheless, incremental gains progressively narrowed at higher intervention levels, suggesting diminishing marginal economic returns once obesity prevalence approaches comparatively low levels.

*Cost-savings from the Greek NHS perspective under varying annual reduction in obesity prevalence thresholds*

When the analysis was restricted to direct healthcare costs only, substantial economic savings remained evident across all examined scenarios, confirming that the projected economic benefits were not driven solely by reductions in productivity losses.

From a healthcare-system perspective, cumulative direct medical savings ranged from EUR 26.16 billion under the 1% annual obesity-reduction scenario to EUR 104.09 billion under the 20% scenario. Correspondingly, discounted savings ranged from EUR 10.52 billion to EUR 47.11 billion.

Under the base-case 5% annual obesity-reduction scenario, cumulative direct healthcare savings were estimated at EUR 59.65 billion, while discounted savings reached EUR 24.49 billion. As in the broader societal-perspective analysis, economic savings increased progressively with the magnitude of obesity reduction, demonstrating a strong, consistent relationship between declining obesity prevalence and reductions in direct healthcare expenditure.

*Indirect cost-savings under varying annual reduction in obesity prevalence thresholds*

In parallel, substantial economic savings were also observed when the analysis focused exclusively on indirect productivity-related costs, highlighting the considerable societal burden obesity imposes through impaired workforce productivity. Cumulative indirect cost-savings ranged from EUR 5.88 billion under the 1% annual obesity-reduction scenario to EUR 17.16 billion under the 20% scenario. Correspondingly, discounted savings ranged from EUR 2.35 billion to EUR 7.82 billion.

Under the base-case 5% annual obesity-reduction scenario, cumulative productivity-related savings were estimated at EUR 11.47 billion, while discounted savings were estimated at EUR 4.77 billion. Although indirect savings remained consistently lower than direct healthcare savings across all examined scenarios, they still accounted for a substantial proportion of the total economic benefit associated with obesity reduction.

*Cost-savings under the 5% base-case annual reduction in obesity prevalence under varying time horizons*

Projected economic savings increased substantially with longer analytical time horizons, indicating that the economic benefits of adult obesity reduction accumulate over time. Under the base-case 5% annual obesity-reduction scenario, cumulative undiscounted cost savings were estimated at EUR 2.88 billion over a 5-year horizon, increasing to EUR 10.08 billion over 10 years, EUR 21.02 billion over 15 years, and EUR 35.16 billion over 20 years. Across the complete 30-year modelling horizon, cumulative undiscounted savings reached EUR 71.12 billion.

A similar pattern emerged in discounted estimates, with discounted savings increasing from EUR 2.41 billion over a 5-year horizon to EUR 7.25 billion over 10 years, EUR 13.03 billion over 15 years, and EUR 18.89 billion over 20 years, ultimately reaching EUR 29.25 billion over the full 30-year projection period.

*Cost-savings under the 5% base-case annual reduction in obesity prevalence under varying discount factors*

The estimated NPV of obesity-related cost savings remained substantial across all examined discount rates, although, as expected, the magnitude of discounted savings progressively declined as higher discount factors were applied.

Discounting strongly influenced the present value of projected economic benefits. Under the hypothetical 5% annual relative reduction in adult obesity prevalence, cumulative discounted gross costs avoided were estimated at EUR 58.68 billion, EUR 48.78 billion, EUR 40.84 billion, and EUR 34.44 billion using 1%, 2%, 3%, and 4% annual discount rates. Higher discount rates reduced the present value of future benefits, with estimates declining to EUR 14.29 billion at 10%, EUR 5.13 billion at 20%, and EUR 2.62 billion at 30%. This pronounced sensitivity reflects the long analytical horizon and the accumulation of a substantial proportion of the modelled economic benefits in later years, which receive progressively less weight as the discount rate increases. The 30% discount rate was examined as an extreme methodological stress test rather than a policy-relevant reference assumption. Accordingly, variation across discount-rate scenarios illustrates how long-term economic valuations depend on assumptions about time preference and the relative importance assigned to benefits accruing to future generations. These estimates represent potential gross costs avoided under the specified prevalence trajectories and should not be interpreted as net economic returns from an evaluated intervention.

*Incremental economic impact of the 5% annual obesity reduction compared with the status quo scenario using crude adult obesity prevalence*

An additional epidemiological scenario analysis examined the implications of replacing the age-standardised obesity-prevalence estimates used in the primary analysis with independently projected crude adult obesity prevalence. Under the crude-prevalence specification, adult obesity prevalence was estimated at 35.83% in 2026 and projected to reach 53.29% by 2055 under the status quo trajectory.

The corresponding annual societal economic burden was estimated at EUR 4.82 billion in 2026, increasing to EUR 5.88 billion by 2055 under the status quo scenario. Under the hypothetical 5% annual relative reduction in adult obesity prevalence, the economic burden was estimated at EUR 4.64 billion in 2026 and EUR 1.33 billion by 2055.

Over the 2026–2055 modelling horizon, the crude-prevalence specification generated cumulative undiscounted gross costs avoided of EUR 80.72 billion and cumulative discounted gross costs avoided of EUR 33.73 billion, applying an annual discount rate of 5%. Compared with the primary analysis based on age-standardised prevalence, which generated EUR 29.25 billion in discounted gross avoided costs, the crude-prevalence scenario yielded approximately 15.32% higher estimate.

#### 3.5.2. Deterministic Sensitivity Analysis

**Figure 3:**
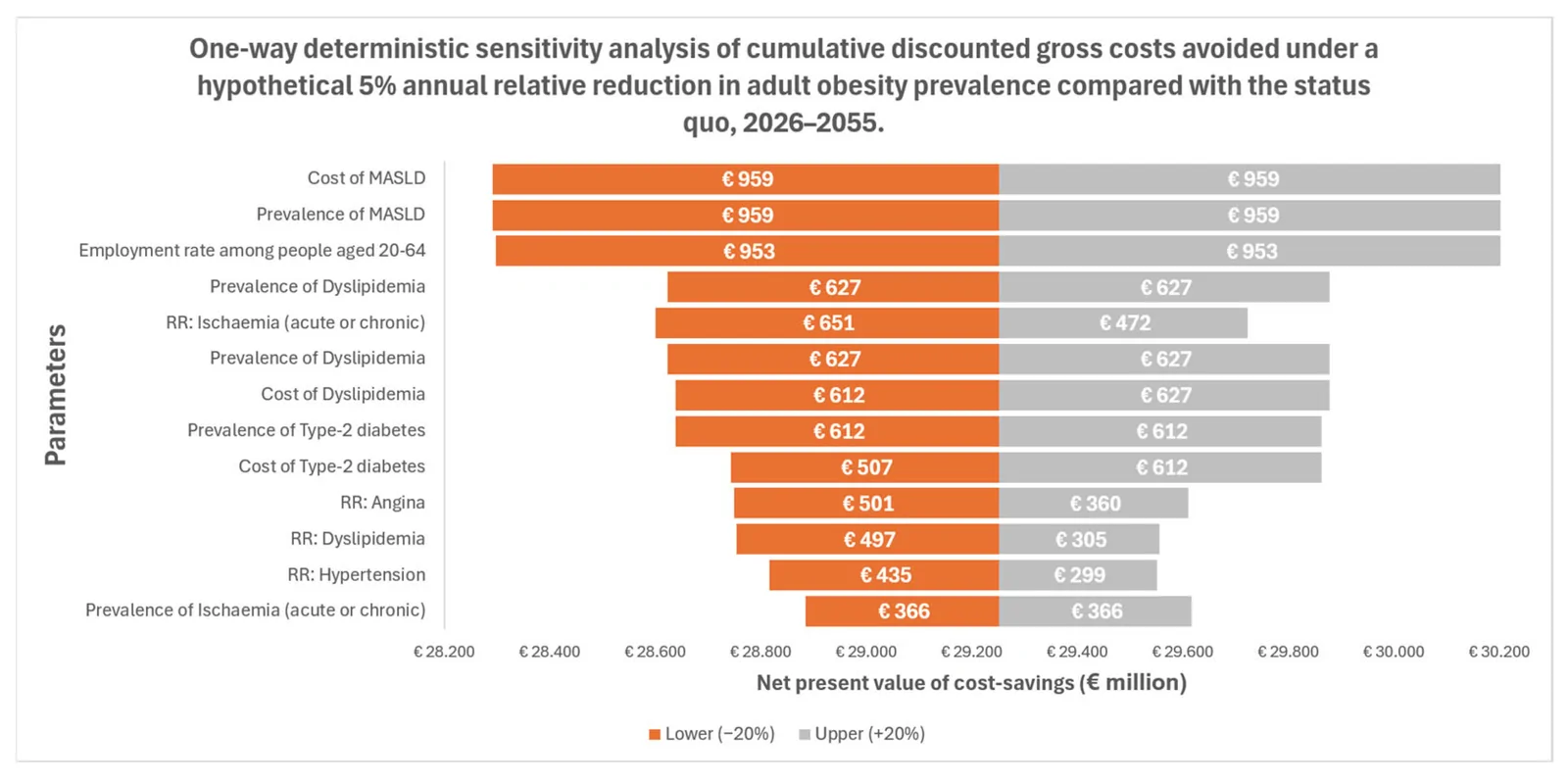
Tornado graph of one-way deterministic sensitivity analysis: Impact of ±20% variation in key parameters on the net present value of cost savings between the 5% annual reduction in obesity prevalence and the future status quo scenario.

The deterministic sensitivity analysis demonstrated that the model outcomes were generally robust to plausible parameter uncertainty, with relatively modest variation around the base-case discounted net present value of EUR 29.25 billion associated with the 5% annual obesity-reduction scenario (Figure 3).

Importantly, the deterministic sensitivity analysis did not incorporate discount rates or baseline obesity prevalence, as we evaluated these separately in dedicated scenario analyses. Specifically, we examined discount factors from 1% to 30% to assess how time preference affects the present valuation of future gross costs avoided, while varying annual obesity-reduction scenarios indirectly captured alternative long-term obesity prevalence trajectories. Their exclusion from the deterministic sensitivity analysis avoided duplication and allowed the one-way sensitivity analysis to focus on uncertainty in epidemiological, clinical, and cost-related model inputs.

Among all examined parameters, the prevalence and cost of MASLD and the employment rate among obese individuals exerted the greatest influence on model outcomes. Varying these parameters by ±20% altered cumulative discounted savings by approximately EUR 950 million under the lower-and-upper-bound scenarios relative to the base case. Similarly, prevalence, relative risk, and cost parameters for type 2 diabetes, dyslipidaemia, angina, ischaemia and hypertension were major drivers of model uncertainty, each resulting in changes in discounted cumulative savings ranging from approximately EUR 299 million to EUR 627 million.

Productivity-related assumptions also moderately influenced the projected economic outcomes. Presenteeism costs associated with BMI class III obesity reduced discounted savings by approximately EUR 291 million, while absenteeism-related costs associated with BMI class III reduced discounted savings by approximately EUR 192 million.

#### 3.5.3. Probabilistic Sensitivity Analysis

The PSA confirmed the robustness and stability of the model projections under simultaneous parameter uncertainty. Across 1000 simulations, the future status quo scenario consistently generated a substantially higher economic burden compared with the 5% annual obesity-reduction scenario, while the intervention scenario maintained considerable economic savings throughout the examined time horizon.

Under the status quo future scenario, the mean cumulative undiscounted obesity-related economic burden was estimated at EUR 137.77 billion, with a standard deviation of EUR 3.87 billion, while the 95% confidence interval ranged from EUR 130.60 billion to EUR 145.06 billion. At a 5% annual discount rate, the mean discounted cumulative burden was estimated at EUR 69.12 billion (95% CI: EUR 65.81–72.49 billion), showing relatively low dispersion and high stability across simulations (Table 4).

Under the 5% annual obesity-reduction scenario, the projected economic burden was substantially lower. Mean cumulative undiscounted costs were estimated at EUR 69.95 billion (SD: EUR 1.75 billion; 95% CI: EUR 66.61–73.54 billion). Discounted mean cumulative costs were estimated at EUR 41.28 billion (95% CI: EUR 39.23–43.44 billion), indicating substantially lower long-term expenditure than in the status quo scenario, even under probabilistic uncertainty.

The incremental economic benefit of the 5% annual reduction in adult obesity prevalence remained consistently positive across all PSA iterations. Mean cumulative undiscounted cost savings were estimated at EUR 67.82 billion (SD: EUR 4.27 billion). The mean discounted NPV of cumulative cost savings was estimated at EUR 27.85 billion (95% CI: EUR 23.83–31.61 billion).

Overall, the PSA shows that the projected long-term economic benefits of sustained reductions in obesity prevalence remain substantial and robust despite uncertainty across epidemiological, clinical, and cost-related model inputs.

## 4. Discussion

This study provides a long-term assessment of the economic burden of adult obesity in Greece and the potential gross costs avoided under alternative population-level obesity-prevalence trajectories. Under the status quo scenario, annual obesity-attributable costs were projected to increase from approximately EUR 4.23 billion in 2025 to EUR 5.11 billion in 2055, despite the anticipated contraction of the adult population. Under the illustrative scenario assuming a sustained 5% annual relative reduction in adult obesity prevalence, cumulative gross costs avoided reached EUR 71.12 billion over 2026–2055, equivalent to EUR 29.25 billion when discounted at 5% annually. These findings demonstrate the substantial economic implications of alternative obesity-prevalence trajectories, although the estimates remain conditional on the model’s epidemiological and economic assumptions and do not represent demonstrated savings from specific interventions.

The economic burden was predominantly driven by obesity-related comorbidities, particularly cardiometabolic and cardiovascular conditions, including type 2 diabetes, dyslipidaemia, hypertension, ischaemic heart disease and MASLD. In contrast, expenditure on obesity pharmacotherapy and bariatric surgery accounted for a comparatively small share of modelled direct medical costs. Productivity losses also contributed substantially to the societal burden, with presenteeism exceeding absenteeism. Together, these findings indicate that the economic consequences of obesity extend beyond its direct management and are largely associated with chronic disease and impaired workforce productivity. Correspondingly, differences in comorbidity-related expenditure and productivity losses accounted for most of the projected gross costs avoided under the alternative prevalence trajectories.

The scenario analyses demonstrated a positive relationship between the magnitude of sustained reductions in obesity prevalence and cumulative economic benefits. However, the assumed 5% annual relative reduction was selected as an illustrative modelling scenario rather than an empirically established or feasible policy target. Over 30 years, this would entail a substantial decline in population obesity prevalence, and the model does not identify the interventions needed to achieve or sustain such a trajectory. Moreover, annual PAF recalculations estimate differences in population-level disease attribution rather than individual disease remission. Consequently, we interpret the projected economic differences as potential changes in obesity-attributable burden under alternative exposure distributions, not as immediately realisable reductions in healthcare expenditure.

The magnitude of the estimated economic benefits should be interpreted in the context of the hypothetical prevalence trajectories underlying the analysis. In the primary scenario, a sustained 5% annual relative reduction in obesity prevalence resulted in a projected prevalence of 6.28% by 2055, compared with 42.87% under the status quo. This substantial divergence is a major determinant of the projected gross costs avoided. Accordingly, these estimates reflect conditional differences in modelled economic burden rather than expected budgetary savings from currently implemented obesity-management programmes. The feasibility, implementation costs and effectiveness of policies capable of producing such sustained population-level changes were not evaluated.

The findings broadly align with international evidence identifying obesity as a major contributor to healthcare expenditure and productivity losses [2,23]. Previous estimates from the World Obesity Federation similarly suggest a considerable and increasing economic burden in Greece, although differences in disease coverage, epidemiological assumptions, costing methodologies, and the inclusion of premature mortality limit direct numerical comparisons [2]. Likewise, economic evaluations of individual weight loss have reported potential reductions in obesity-related healthcare costs and productivity losses [18,19,20,21]. However, these studies typically examine changes in individual body weight, whereas the present analysis models changes in population obesity prevalence. These outcomes are conceptually and methodologically distinct, and findings from individual-level weight-loss studies cannot directly validate the magnitude of the economic differences estimated here. Nevertheless, the prominence of cardiometabolic disease and productivity losses across studies supports the broader economic relevance of obesity-related morbidity.

A key contribution of this study is integrating national demographic projections, long-term obesity-prevalence forecasting, 36 obesity-related comorbidities, and direct and indirect economic costs into a unified population-level framework. The inclusion of a broad disease spectrum, including MASLD, mental health conditions and obesity-associated malignancies, extends the scope of several earlier modelling studies focused primarily on cardiometabolic outcomes. Furthermore, combining deterministic, probabilistic, and scenario analyses provides a structured assessment of uncertainty under alternative epidemiological and economic assumptions. However, the probabilistic estimate of EUR 27.85 billion in discounted gross costs avoided, with a 95% uncertainty interval of EUR 23.83–31.61 billion, should be interpreted as conditional on the selected parameter distributions and model structure rather than as encompassing all sources of uncertainty.

From a policy perspective, the results illustrate the potential long-term economic importance of reducing population obesity prevalence and the relevance of considering both healthcare expenditure and productivity losses in public health planning. Nevertheless, this analysis is not an intervention-specific cost-effectiveness, cost–benefit, or budget impact evaluation. It does not estimate the effectiveness, implementation costs, affordability or sustainability of the interventions required to achieve the assumed prevalence reductions. It also does not assess health-system capacity or the distributional consequences of alternative policies. Accordingly, the projected economic differences represent potential gross costs avoided rather than net savings or returns on investment. Intervention-specific economic evaluations, supported by evidence on clinical effectiveness, uptake, implementation requirements and equity, are needed to inform the selection and prioritisation of obesity-management strategies.

Several methodological limitations warrant consideration. First, the model relied partly on epidemiological and economic inputs derived from non-Greek populations. Although national evidence was prioritised where available, differences in population characteristics, healthcare utilisation and costing practices may limit parameter transferability. This is particularly relevant to productivity estimates, which we adjusted using relative GDP per capita and the Greek employment rate, but we could not fully capture national differences in occupational characteristics, informal employment, sick-leave arrangements, or presenteeism. In addition, selecting lower estimates from available sources does not necessarily eliminate uncertainty or ensure conservative results. The assumed stability of BMI-class distributions, employment rates, disease-specific relative risks and real unit costs throughout the projection horizon further limits the model’s ability to capture future changes in epidemiological and economic conditions.

Second, an important methodological consideration concerns the use of age-standardised rather than crude obesity-prevalence estimates in the primary analysis. Although age-standardised prevalence facilitates comparisons over time by accounting for differences in population age structure, applying it to the adult population may introduce uncertainty into estimates of absolute obesity burden and associated economic costs. To address this, we ran a separate scenario analysis using crude adult obesity prevalence, aligning the prevalence measure more directly with the population denominator. This alternative specification generated cumulative discounted gross costs avoided of EUR 38.28 billion, compared with EUR 33.44 billion in the primary analysis, representing an increase of approximately 14.5%. Although the economic findings remained consistent, the difference shows that the choice of epidemiological prevalence measure meaningfully affects the magnitude of projected economic outcomes. The crude-prevalence scenario therefore provides an important assessment of structural uncertainty and reinforces the potential economic value associated with sustained reductions in adult obesity prevalence. However, retaining age-standardised prevalence in the primary analysis represents a structural limitation because these estimates are not directly interchangeable with crude prevalence when applied to actual adult population counts. The model also does not explicitly account for age-specific differences in obesity prevalence, comorbidity risks, or healthcare expenditure, which future research should consider when incorporating age-stratified epidemiological and demographic projections.

Third, further methodological consideration concerns estimating obesity-attributable productivity losses. To ensure consistency with the age range underlying the employment-rate input, the population eligible to generate absenteeism and presenteeism costs was restricted to individuals aged 20–64 years. This adjustment avoids applying a working-age employment rate to the entire adult population, including individuals aged 65 years and older. Nevertheless, because age-specific obesity-prevalence estimates were unavailable, we used overall adult obesity prevalence as a proxy for the working-age population. Furthermore, the general population employment rate was assumed to be representative of individuals living with obesity, although employment participation may differ by obesity status. These assumptions introduce residual uncertainty into the estimated productivity burden. Future research incorporating age-specific obesity prevalence, employment participation by BMI category and longitudinal demographic projections could improve the precision of these estimates.

Fourth, the prevalence-based PAF framework does not explicitly model disease onset, progression, remission, latency, recurrent events or residual risk following changes in obesity status. Consequently, obesity-attributable burden may change more gradually than the annual attribution approach implies, potentially affecting both the timing and magnitude of projected economic differences. Furthermore, independently aggregating disease-specific costs does not explicitly account for multimorbidity, overlapping healthcare resource utilisation, or competing mortality risks, introducing a risk of double counting. Correlations between epidemiological and economic parameters were not explicitly represented in the probabilistic analysis, while alternative structural specifications were not evaluated. The reported uncertainty intervals therefore do not capture the full extent of structural and forecasting uncertainty.

Fifth, a further structural limitation arises from the assumption that changes in population obesity prevalence are reflected contemporaneously in obesity-attributable comorbidity burden and associated healthcare costs. In clinical practice, the relationship between obesity reduction and disease outcomes is more complex. Some complications may persist despite subsequent reductions in body weight, whereas other outcomes may improve only after prolonged exposure to a lower-risk metabolic state. Because the model does not explicitly incorporate disease latency, progression, remission, mortality or competing risks, it cannot determine when reductions in healthcare utilisation and expenditure would materialise. Over the 30-year projection horizon, this simplification may overstate the immediacy of economic benefits and alter their cumulative discounted value. Furthermore, the aggregate costing framework does not explicitly account for multimorbidity or overlapping expenditure among individuals with concurrent obesity-related conditions, introducing a risk of double counting. Consequently, the projected gross costs avoided should be interpreted as comparative, model-derived estimates rather than directly realisable reductions in healthcare expenditure.

Sixth, the economic estimates also face uncertainty due to the availability and transferability of cost inputs. For several obesity-related conditions, Greece-specific estimates were unavailable, so we adapted evidence from other healthcare systems. Although monetary values were adjusted to the Greek economic context and the common 2025 price year, differences in clinical practice, resource utilisation and reimbursement arrangements may not be fully captured. Similarly, we estimated productivity losses using internationally derived BMI-specific cost estimates and assumptions about employment participation, rather than directly observed Greece-specific productivity data. The model included pharmacotherapy and bariatric surgery costs only for the treatment categories and financing arrangements represented, and did not provide a comprehensive assessment of the costs of achieving the hypothetical prevalence reductions. These limitations affect the interpretation of the absolute economic estimates and reinforce the distinction between modelled gross costs avoided and the net economic value of specific interventions.

Finally, we necessarily simplified the representation of obesity-management expenditure. Pharmacotherapy costs were based on liraglutide, with fixed assumptions about treatment eligibility, uptake, persistence, and pack utilisation, without explicitly modelling switching, reinitiation, or confidential rebates. Other anti-obesity pharmacotherapies financed through temporary funding arrangements were excluded, while bariatric surgery costs were based on assumed uptake and reimbursement tariffs without modelling longer-term postoperative expenditure. The analysis also did not comprehensively capture direct non-medical costs, out-of-pocket expenditure, informal care, premature mortality or paediatric obesity. The study excluded health-related quality of life, quality-adjusted life-years, and equity outcomes. These exclusions limit the comprehensiveness of the estimated economic and health burden, although their combined influence on the differences between scenarios cannot be established from the present analysis.

Overall, the study indicates that adult obesity may impose a substantial and increasing economic burden on Greece, while sustained reductions in population obesity prevalence could be associated with considerable long-term gross costs avoided under the specified modelling assumptions. These findings provide a quantitative framework for understanding the potential economic consequences of alternative epidemiological trajectories, but do not establish the feasibility or net economic value of interventions. Future research should integrate age-specific epidemiological data, longitudinal disease dynamics and intervention-specific evidence to evaluate the effectiveness, affordability, equity and long-term economic consequences of obesity-prevention and management policies [132,133,134,135].

## 5. Conclusions

Adult obesity is projected to impose a substantial and increasing healthcare and societal economic burden in Greece over the coming decades. This analysis shows that sustained reductions in population obesity prevalence could avoid considerable cumulative gross costs relative to the projected status quo. However, the magnitude of these estimates depends on the hypothetical prevalence trajectories and the model’s epidemiological, economic, and structural assumptions. In particular, the absence of explicit modelling of disease progression, latency, mortality, and multimorbidity limits the interpretation of projected economic differences as realisable healthcare savings. Accordingly, these findings should be considered illustrative estimates of the potential economic consequences of alternative obesity-prevalence trajectories, rather than evidence of the feasibility, affordability or cost-effectiveness of specific obesity-management interventions. Future research incorporating longitudinal disease dynamics, age-specific epidemiological data and Greece-specific economic evidence is warranted to support more comprehensive policy evaluations.

## Figures and Tables

**Table 1:** Obesity-attributed comorbidities using the Population Attributable Fraction framework.

Comorbidity	Prevalence (%)	Patients in Greece	Relative Risk	PAF (%)	Patients Due to Obesity
Angina	2.50% [59]	217,714	1.88 [60]	20.47%	44,560
Atrial fibrillation	1.67% [61]	145,498	1.5 [62]	12.76%	18,560
Congestive heart failure	1.00% [63]	87,086	2.00 [62]	22.63%	19,704
Hypertension	17.70% [59]	1,541,414	2.17 [60]	25.49%	392,945
Ischaemia (acute or chronic)	2.02% [64]	175,564	1.81 [65]	19.15%	33,622
Myocardial infarction	2.00% [59]	174,171	1.17 [60]	4.74%	8249
Pulmonary embolism	0.03% [66]	2473	2.32 [67]	27.85%	689
Stroke	0.42% [68]	36,656	1.50 [62]	12.76%	4676
Dyslipidemia	16.70% [69]	1,454,329	2.86 [70]	35.23%	512,360
Type-2 diabetes	7.40% [71]	644,433	4.56 [72]	51.01%	328,699
Polycystic Ovary Syndrome (PCOS) ^1^	6.77% [73]	300,091	1.95 [74]	21.74%	65,243
Obstructive Sleep Apnea	1.00% [75]	87,086	7.18 [60]	64.38%	56,064
Asthma	9.10% [76]	792,479	1.92 [77]	21.20%	168,007
Osteoarthritis	0.90% [78]	78,377	4.55 [79]	50.94%	39,922
Depression	2.90% [80]	252,548	1.15 [81]	4.20%	10,612
Anxiety	4.10% [80]	357,051	2.1 [82]	24.34%	86,901
Dementia	1.39% [83]	121,248	1.36 [84]	9.52%	11,549
Gastroesophageal Reflux Disease	7.06% [85]	614,650	1.76 [86]	18.18%	111,765
Chronic Kidney Disease	7.21% [87]	627,887	1.81 [88]	19.15%	120,245
Breast cancer ^1^	0.41% [89]	35,740	1.26 [90]	7.04%	2525
Colorectal cancer	0.26% [89]	22,230	1.31 [91]	8.28%	1848
Kidney cancer	0.07% [89]	5948	1.77 [92]	18.32%	1093
Leukaemia	0.07% [89]	5846	1.11 [93]	3.10%	182
Liver cancer	0.02% [89]	1985	1.89 [94]	20.58%	410
Oesophagal cancer	0.001% [89]	365	2.6 [95]	31.78%	116
Ovarian cancer ^1^	0.03% [89]	2607	1.1 [96]	2.83%	74
Pancreatic cancer	0.02% [89]	1895	1.19 [97]	5.24%	100
Prostate cancer ^1^	0.36% [89]	31,082	1.13 [98]	3.65%	1138
Thyroid cancer	0.05% [89]	4077	1.54 [99]	13.59%	556
Endometrial cancer ^1^	0.01% [89]	900	2.1 [100]	24.26%	219
Gallbladder Cancer	0.001% [89]	84	1.1 [101]	2.83%	2
Multiple myeloma	0.03% [89]	2511	1.2 [102]	5.50%	139
Stomach cancer	0.01% [89]	2654	1.1 [103]	2.83%	75
MASLD ^2^	9.74% [104]	848,205	3.53 [105]	42.52%	360,690
Gout	4.71% [106]	410,173	2.24 [107]	26.61%	109,154
Gallstones/Cholelithiasis	5.20% [108]	452,845	2.00 [109]	22.63%	102,463

^1^ For sex-specific conditions, published national case counts were used directly when available. Sex-specific population proportions were applied only when deriving case counts from prevalence rates. GLOBOCAN 5-year prevalent cancer counts were not subjected to additional sex adjustment. ^2^ The modelled prevalence represents 17.3% of the population-based MASLD prevalence multiplied by the 56.3% proportion of individuals with MASLD who have elevated metabolic markers consistent with steatohepatitis risk. This subgroup is not equivalent to histologically confirmed MASH.

**Table 2:** Annual direct medical and indirect productivity costs per patient (costs adjusted to 2025 euros).

Disease	Original Cost Value (EUR)	Adjusted Cost Value (EUR)	Reference
Obesity-Related Complications Cost			
Angina	EUR 3715.00	EUR 4524.91	Gourzoulidis et al. [110]
Atrial fibrillation	EUR 2947.00	EUR 3756.37	Athanasakis et al. [111]
Congestive heart failure	EUR 3910,00	EUR 4580.21	Stafylas et al. [112]
Hypertension	EUR 468.44	EUR 604.32	Tsalta et al. [113]
Ischaemia (acute or chronic)	EUR 5875.00	EUR 7155.82	Gourzoulidis et al. [110]
Myocardial infarction	EUR 6170.00	EUR 7515.13	Gourzoulidis et al. [110]
Pulmonary embolism	EUR 1071.00	EUR 2495.40	Gourzoulidis et al. [110]
Stroke	EUR 5714.83	EUR 6826.17	Siskou et al. [114]
Dyslipidemia	EUR 778.60	EUR 940.51	Migdalis et al. [115]
Type-2 diabetes	EUR 1297.30	EUR 1743.10	Athanasakis et al. [116]
Polycystic Ovary Syndrome (PCOS)	EUR 583.96	EUR 706.06	Riestenberg et al. [117]
Obstructive Sleep Apnea	EUR 1331.86	EUR 1718.19	Prapa, Geitona, & Gourgoulianis. [118]
Asthma	EUR 673.00	EUR 810.63	Vellopoulou et al. [119]
Osteoarthritis	EUR 813.40	EUR 971.58	Savvari et al. [120]
Depression	EUR 154.50	EUR 180.98	Tzanetakos et al. [121]
Anxiety	EUR 77.25	EUR 90.49	Half of depression
Dementia	EUR 1311.48	EUR 1565.90	Tsampalas et al. [122]
Gastroesophageal Reflux Disease	EUR 563.00	EUR 659.50	Varannes et al. [123]
Chronic Kidney Disease	EUR 758.01	EUR 825.77	Stafylas et al. [74] & Koulentaki et al. [124]
Cancer (all types, average)	EUR 13,976.05	EUR 16,729.58	Hofmarcher et al. [125] & WHO [126]
MASLD	EUR 1860	EUR 2026.27	Ossima et al. [127]
Gout	EUR 1337.00	EUR 1456.52	Rotaru et al. [128]
Gallstones/Cholelithiasis	EUR 587.30	EUR 701.51	Fanourgiakis et al. [129]
Indirect costs			
BMI class	Adjusted absenteeism cost per capita (EUR)	Adjusted presenteeism cost per capita (EUR)	
30–34.9	EUR 77,50	EUR 74,30	Gupta et al. [48]
35–39.9	EUR 249,15	EUR 223,53	Gupta et al. [48]
>40	EUR 514,95	EUR 780,76	Gupta et al. [48]

**Table 3:** Total obesity-attributable costs in Greece, 2025 (in millions of EUR).

Cost Parameter	Total Cost (EUR Million)	% of Total Cost
Direct Medical Costs		
Comorbidities	EUR 3682.11	87.05%
Bariatric surgery	EUR 5.77	0.14%
Pharmacotherapy	EUR 3.24	0.08%
Total direct costs	EUR 3691.11	87.26%
Indirect costs		
Absenteeism	EUR 246.75	5.83%
Presenteeism	EUR 291.97	6.90%
Total indirect costs	EUR 538.72	12.74%
Total costs	EUR 4229.83	100.00%

**Table 4:** Probabilistic sensitivity analysis of cumulative undiscounted and discounted obesity-related costs and economic savings under the status quo and base-case 5% annual obesity-reduction scenarios (values are in EUR millions).

Scenarios	Mean	Median	Standard Deviation	Minimum	Maximum	95% CI Lower	95% CI Upper
Status Quo-Future Scenario							
Total undiscounted costs	EUR 137,769	EUR 137,774	EUR 3856	EUR 126,829	EUR 152,098	EUR 130,601	EUR 145,057
Total discounted costs	EUR 69,128	EUR 69,129	EUR 1720	EUR 63,993	EUR 74,899	EUR 65,809	EUR 72,496
5% annual adult obesity prevalence reduction							
Total undiscounted costs	EUR 69,954	EUR 69,948	EUR 1751	EUR 64,381	EUR 74,948	EUR 66,610	EUR 73,537
Total discounted costs	EUR 41,279	EUR 41,275	EUR 1085	EUR 38,146	EUR 44,528	EUR 39,226	EUR 43,443
Incremental economic benefit							
Total undiscounted cost savings	EUR 67,816	EUR 67,860	EUR 4276	EUR 53,786	EUR 85,153	EUR 59,217	EUR 75,864
Total discounted cost savings (Net Present Value)	EUR 27,849	EUR 27,864	EUR 2018	EUR 20,703	EUR 35,486	EUR 23,826	EUR 31,612

## Data Availability

The economic model developed in Microsoft Excel is available upon reasonable request from the corresponding author. The reason for restricted access is not related to any confidentiality or ethical restrictions on the underlying data. Rather, the model is a researcher-developed analytical tool containing detailed calculations, assumptions, model structure, and linked formulas developed specifically for this study.

## Supplementary Materials

The following supporting information can be downloaded at: https://www.mdpi.com/article/10.3390/healthcare14213578/s1, Table S1. Model calculation framework; Table S2. Principal epidemiological, clinical and economic input parameters; Table S3. Adult obesity forecasting (age-standardised values); Table S4. Adult obesity forecasting (crude values); Table S5. Annual calculation and discounting audit trail; Figure S1. Forecasted cost of adult obesity under the future status quo scenario; Figure S2. Forecasted cost of adult obesity under the 5% annual obesity reduction scenario; Figure S3. Forecasted cost savings associated with the comparison of the status quo versus the 5% annual obesity reduction scenario.

## Abbreviations

The following abbreviations are used in this manuscript:

BMI	Body Mass Index
CHD	Coronary Heart Disease
CI	Confidence Interval
COI	Cost of Illness
CPI	Consumer Price Index
CPRD	Clinical Practice Research Datalink
CrI	Credible Interval
CV	Coefficient of Variation
CVD	Cardiovascular Disease
DSA	Deterministic Sensitivity Analysis
ELSTAT	Hellenic Statistical Authority
EOPYY	National Organization for the Provision of Health Services
EQ-5D	EuroQol 5-Dimension
ETS	Exponential Smoothing
GDP	Gross Domestic Product
GERD	Gastroesophageal Reflux Disease
GIP	Glucose-Dependent Insulinotropic Polypeptide
GLP-1	Glucagon-Like Peptide-1
HR	Hazard Ratio
HRQoL	Health-Related Quality of Life
IMF	International Monetary Fund
IQR	Interquartile Range
IRR	Incidence Rate Ratio
MI	Myocardial Infarction
MASLD	Metabolic dysfunction-associated steatotic liver disease
NASH	Non-Alcoholic Steatohepatitis
NCD	Non-Communicable Disease
NPV	Net Present Value
OECD	Organisation for Economic Co-operation and Development
ORC	Obesity-Related Comorbidity
ORCs	Obesity-Related Comorbidities
OSA	Obstructive Sleep Apnoea
PAF	Population-Attributable Fraction
PCOS	Polycystic Ovary Syndrome
PSA	Probabilistic Sensitivity Analysis
QALY	Quality-Adjusted Life-Year
RR	Relative Risk
RRF	Recovery and Resilience Facility
SF-36	36-Item Short Form Health Survey
T2D	Type 2 Diabetes Mellitus
UK	United Kingdom
USA	United States of America
WHO	World Health Organization
