# Supplementary material for: The Economic Value of Reducing Adult Obesity in Greece: A Prevalence-Based Dynamic Modelling Study
Source: Healthcare (Basel). 2026 Oct 22;14(21):3578. doi: 10.3390/healthcare14213578 (PMC13762492; doi:10.3390/healthcare14213578)
Supplement: Supplementary file 1 [file healthcare-14-03578-s001.zip › healthcare-4580809-supplementary.pdf]

Supplementary Table S1: Model calculation framework

Model component	Parameter / outcome	Mathematical specification	Description and assumptions
Population projection	Adult population in year t	$N_{AD,t}=ETS(N_{AD,historical})$	Adult population projected using exponential smoothing based on historical demographic observations.
Obesity prevalence	Obesity prevalence under status quo	$P_{OB,SQ,t}=ETS(P_{OB,historical})$	Historical WHO obesity-prevalence series extrapolated to 2055 using a non-seasonal ETS forecasting specification.
Obesity prevalence reduction	Prevalence under scenario s	$P_{OB,s,t}=P_{OB,2025}(1-s)^{t-2025}$	Hypothetical annual relative reduction in adult obesity prevalence; s represents the annual reduction rate (e.g., 0.05 for 5%). Verify the exact recurrence against the final workbook.
Population with obesity	Adults living with obesity	$N_{OB,s,t}=N_{AD,t}P_{OB,s,t}$	Annual number of adults with obesity under each scenario.
Disease-specific population	Population eligible for disease i	$N_{ELIG,i,t}=N_{AD,t}f_{i,t}$	Applies relevant sex or age eligibility where necessary, e.g., women for PCOS.
Comorbidity prevalence	Population with comorbidity i	$N_{DIS,i,t}=N_{ELIG,i,t}P_{DIS,i}$	Disease-specific prevalence applied to the relevant population. Disease prevalence assumed constant unless otherwise specified.
Population-attributable fraction	Obesity-attributable fraction for disease i	$PAF_{i,s,t}=\frac{P_{OB,s,t}(RR_i-1)}{1+P_{OB,s,t}(RR_i-1)}$	Standard PAF formulation based on obesity prevalence and disease-specific relative risk.
Obesity-attributable cases	Cases attributable to obesity	$N_{ATTR,i,s,t}=N_{DIS,i,t}PAF_{i,s,t}$	Estimated population-level attributable cases; not an individual-level prediction of disease remission.
Comorbidity-related costs	Annual cost of disease i	$C_{DIS,i,s,t}=N_{ATTR,i,s,t}UC_{i,2025}$	Obesity-attributable cases multiplied by annual disease-specific unit costs, expressed in 2025 euros.
Total comorbidity costs	Aggregate ORC expenditure	$C_{ORC,s,t}=\sum_i C_{DIS,i,s,t}$	Summation across all 36 included obesity-related comorbidities.
Obesity pharmacotherapy	Annual pharmacotherapy expenditure	$C_{PHARM,s,t}=N_{TREATED,s,t}UC_{PHARM}$	Eligible and treated population multiplied by annual treatment acquisition cost. Treatment eligibility and uptake are model inputs.

Bariatric surgery	Annual surgical expenditure	($C_{SURG,s,t}=N_{SURG,s,t}UC_{SURG}$)	Number of procedures multiplied by unit procedure cost.
Direct medical costs	Total annual direct costs	($C_{DIRECT,s,t}=C_{ORC,s,t}+C_{PHARM,s,t}+C_{SURG,s,t}$)	Sum of comorbidity-related expenditure and obesity-management costs.
BMI class distribution	Adults with obesity by BMI class	($N_{OB,j,s,t}=N_{OB,s,t}w_j$)	Adults with obesity distributed across BMI classes I, II and III using fixed class proportions.
Employed population	Employed adults with obesity by BMI class	($N_{EMP,j,s,t}=N_{OB,j,s,t}ER_t$)	Employment-rate adjustment for workplace productivity losses. The final workbook must confirm whether the working-age denominator is applied separately.
Absenteeism	Annual absenteeism-related costs	($C_{ABS,s,t}=\sum_j N_{EMP,j,s,t}UC_{ABS,j}$)	Employment-adjusted number of adults with obesity multiplied by BMI-class-specific absenteeism costs.
Presenteeism	Annual presenteeism-related costs	($C_{PRES,s,t}=\sum_j N_{EMP,j,s,t}UC_{PRES,j}$)	Employment-adjusted number of adults with obesity multiplied by BMI-class-specific presenteeism costs.
Indirect costs	Total productivity losses	($C_{INDIRECT,s,t}= C_{ABS,s,t}+C_{PRES,s,t}$)	Total annual productivity-related economic burden.
Societal burden	Total annual obesity-attributable costs	($C_{TOTAL,s,t}=C_{DIRECT,s,t}+C_{INDIRECT,s,t}$)	Annual economic burden from the societal perspective.
Scenario comparison	Potential gross costs avoided	($\Delta C_{s,t}=C_{TOTAL,SQ,t}-C_{TOTAL,s,t}$)	Annual difference in economic burden between status quo and the hypothetical prevalence-reduction scenario.
Cumulative undiscounted costs avoided	Total gross costs avoided	($G_s=\sum_{t=2026}^{2055}\Delta C_{s,t}$)	Sum of annual undiscounted cost differences over the 30-year horizon.
Discounting	Annual discounted gross costs avoided	($DC_{s,t}=\frac{\Delta C_{s,t}}{(1+r)^{t-2025}}$)	Annual cost differences discounted to the 2025 base year.
Net present value	Cumulative discounted gross costs avoided	($NPV_s=\sum_{t=2026}^{2055}DC_{s,t}$)	Present value of potential gross costs avoided; does not represent net savings or return on investment.

Abbreviations: ETS, exponential smoothing state-space model; PAF, population-attributable fraction; RR, relative risk; ORC, obesity-related comorbidity; BMI, body mass index; NPV, net present value; SQ, status quo; UC, unit cost; ER, employment

rate. Subscripts: \(i\), comorbidity; \(j\), BMI class; \(s\), scenario; \(t\), calendar year. These are mathematical representations of the manuscript's described framework; verify exact implementation details against the final Excel model.

Supplementary Table S2. Principal epidemiological, clinical and economic input parameters

Parameter	Base-case value / specification	Source or methodological basis	Uncertainty / scenario analysis
Country	Greece	Study setting	Fixed
Target population	Adults aged ≥18 years	Study population	Fixed
Base year	2025	Model specification	Fixed
Projection horizon	2026–2055	Model specification	30 years
Economic perspective	Societal	Study methodology	Direct and indirect costs reported separately
Currency and price year	Euro (€), 2025	ELSTAT CPI adjustments	Fixed price year
Adult population, 2025	8,708,555	ELSTAT / demographic inputs	Updated annually through ETS projections
Adult population forecasting	Non-seasonal ETS	Historical Greek demographic series	Forecast uncertainty incorporated into sensitivity analyses
Adult obesity prevalence, 2025	29.24%	WHO GHO series and ETS projection	Forecast uncertainty examined
Obesity prevalence forecasting	Non-seasonal ETS	WHO historical obesity prevalence	Lower and upper forecast bounds
Status quo obesity prevalence, 2055	42.87%	ETS projection	34.91%–50.83% forecast interval
Annual relative prevalence-reduction scenarios	1%–20%	Hypothetical scenario assumptions	Multiple annual reduction rates
Primary illustrative scenario	5% annual relative reduction	Selected modelling reference scenario; not an intervention-effectiveness estimate	Alternative reduction scenarios
Obesity prevalence, 2055, under 5% scenario	6.28%	Model-derived value	Recalculate if epidemiological inputs change

Number of obesity-related comorbidities	36	Modelled disease set	Disease-specific attribution and costing
Comorbidity prevalence	Disease-specific	National and international epidemiological sources	Parameter-specific uncertainty where available
Relative risk of comorbidity associated with obesity	Disease-specific	Published epidemiological studies, preferably systematic reviews and meta-analyses	Disease-specific estimates; constant over time in the base model
Population-attributable fraction	Calculated annually by disease and scenario	Standard PAF equation	Varies with obesity prevalence and RR assumptions
Female population proportion	50.9%	Population demographic input	Relevant to sex-specific conditions
MASLD prevalence	17.3% overall; 9.74% derived subgroup	Greek population-based epidemiological study	Verify costed subgroup definition
Disease-specific annual treatment costs	Condition-specific, in 2025 euros	Greek economic studies and other published sources	Unit-cost uncertainty
Obesity pharmacotherapy	Eligible treated patients × annual drug cost	Greek reimbursement and treatment assumptions	Uptake and cost assumptions
Bariatric surgery	Number of procedures × unit procedure cost	Greek utilisation and reimbursement assumptions	Uptake and unit-cost assumptions
BMI class I proportion	56.32%	BMI-class distribution source cited in manuscript	Fixed distribution unless varied
BMI class II proportion	26.81%	BMI-class distribution source cited in manuscript	Fixed distribution unless varied
BMI class III proportion	16.86%	BMI-class distribution source cited in manuscript	Fixed distribution unless varied
Employment rate	71.0%	Eurostat, Greek population aged 20–64 years	Employment uncertainty examined
Population aged 20-64/adult population	69,17%	ELSTAT	Uncertainty examined
Annual absenteeism cost, BMI class I	€77.50	Gupta et al., adjusted to Greece and 2025 euros	Unit-cost uncertainty

Annual absenteeism cost, BMI class II	€249.15	Gupta et al., adjusted to Greece and 2025 euros	Unit-cost uncertainty
Annual absenteeism cost, BMI class III	€514.95	Gupta et al., adjusted to Greece and 2025 euros	Unit-cost uncertainty
Annual presenteeism cost, BMI class I	€74.30	Gupta et al., adjusted to Greece and 2025 euros	Unit-cost uncertainty
Annual presenteeism cost, BMI class II	€223.53	Gupta et al., adjusted to Greece and 2025 euros	Unit-cost uncertainty
Annual presenteeism cost, BMI class III	€780.76	Gupta et al., adjusted to Greece and 2025 euros	Unit-cost uncertainty
Base-case annual discount rate	5%	Study assumption	Alternative discount rates
Sensitivity analyses	Deterministic, probabilistic and scenario analyses	Model methodology	Parameter and scenario uncertainty
Costs of implementing population-level obesity policies	Not modelled	Outside study scope	No intervention-specific cost-effectiveness analysis
Premature mortality costs	Not included	Data and methodological limitations	Outside study scope
Individual disease remission and progression	Not explicitly modelled	Prevalence-based PAF framework	Structural limitation

Values reflect those reported or discussed in the revised manuscript and subsequent methodological revisions.

Supplementary Table S3. Adult obesity forecasting (age-standarized values)

Years	Historical values	Forecasted values	Lower limit	Upper limit
1980	12,03			
1981	12,14			
1982	12,26			
1983	12,39			
1984	12,52			
1985	12,66			
1986	12,8			
1987	12,96			
1988	13,13			
1989	13,31			
1990	13,52			
1991	13,76			

1992	14,03			
1993	14,33			
1994	14,68			
1995	15,06			
1996	15,49			
1997	15,95			
1998	16,45			
1999	16,98			
2000	17,54			
2001	18,12			
2002	18,73			
2003	19,34			
2004	19,97			
2005	20,6			
2006	21,22			
2007	21,82			
2008	22,39			
2009	22,91			
2010	23,39			
2011	23,82			
2012	24,2			
2013	24,56			
2014	24,9			
2015	25,21			
2016	25,51			
2017	25,79			
2018	26,04			
2019	26,28			
2020	26,51			
2021	26,74			
2022	26,97			
2023	27,2			
2024	27,43			
2025		29,24	29,09	29,40
2026		29,70	29,48	29,92
2027		30,15	29,83	30,47
2028		30,61	30,16	31,05
2029		31,06	30,47	31,65
2030		31,51	30,76	32,27
2031		31,97	31,03	32,90
2032		32,42	31,30	33,54
2033		32,88	31,55	34,20
2034		33,33	31,79	34,87
2035		33,78	32,02	35,54
2036		34,24	32,24	36,23
2037		34,69	32,46	36,93

2038		35,15	32,66	37,64
2039		35,60	32,85	38,35
2040		36,05	33,03	39,07
2041		36,51	33,21	39,81
2042		36,96	33,38	40,55
2043		37,42	33,54	41,30
2044		37,87	33,69	42,05
2045		38,33	33,84	42,81
2046		38,78	33,97	43,59
2047		39,23	34,10	44,36
2048		39,69	34,23	45,15
2049		40,14	34,34	45,94
2050		40,60	34,45	46,74
2051		41,05	34,56	47,54
2052		41,50	34,65	48,35
2053		41,96	34,74	49,17
2054		42,41	34,83	50,00
2055		42,87	34,91	50,83

Supplementary Table S4. Adult obesity forecasting (crude values)

Years	Historical values	Forecasted values	Lower limit	Upper limit
1980	13,3			
1981	13,42			
1982	13,58			
1983	13,73			
1984	13,89			
1985	14,05			
1986	14,23			
1987	14,41			
1988	14,61			
1989	14,84			
1990	15,1			
1991	15,39			
1992	15,69			
1993	16,01			
1994	16,38			
1995	16,8			
1996	17,27			
1997	17,77			
1998	18,31			
1999	18,89			
2000	19,51			
2001	20,17			
2002	20,87			
2003	21,62			

2004	22,38			
2005	23,16			
2006	23,94			
2007	24,72			
2008	25,46			
2009	26,17			
2010	26,84			
2011	27,49			
2012	28,12			
2013	28,71			
2014	29,29			
2015	29,83			
2016	30,34			
2017	30,78			
2018	31,19			
2019	31,57			
2020	31,94			
2021	32,42			
2022	32,89			
2023	33,26			
2024	33,69			
2025		35,23	35,08	35,39
2026		35,83	35,56	36,11
2027		36,44	35,99	36,88
2028		37,04	36,40	37,68
2029		37,64	36,78	38,50
2030		38,24	37,14	39,35
2031		38,84	37,47	40,21
2032		39,45	37,79	41,10
2033		40,05	38,09	42,00
2034		40,65	38,38	42,92
2035		41,25	38,65	43,85
2036		41,85	38,91	44,80
2037		42,45	39,15	45,76
2038		43,06	39,38	46,74
2039		43,66	39,59	47,73
2040		44,26	39,79	48,73
2041		44,86	39,98	49,74
2042		45,46	40,16	50,77
2043		46,07	40,33	51,80
2044		46,67	40,48	52,85
2045		47,27	40,62	53,91
2046		47,87	40,76	54,98
2047		48,47	40,88	56,07
2048		49,07	40,99	57,16
2049		49,68	41,09	58,26

2050		50,28	41,19	59,37
2051		50,88	41,27	60,49
2052		51,48	41,34	61,62
2053		52,08	41,40	62,76
2054		52,69	41,46	63,91
2055		53,29	41,50	65,07

Supplementary Table S5. Annual calculation and discounting audit trail

Year	Status quo total costs	5% scenario total costs	Annual gross costs avoided	Discount factor (5%)	Discounted gross costs avoided
2026	€ 4.266,50	€ 4.067,82	€ 198,68	0,95238095	€ 189,22
2027	€ 4.302,62	€ 3.910,51	€ 392,11	0,90702948	€ 355,65
2028	€ 4.338,19	€ 3.757,84	€ 580,35	0,8638376	€ 501,33
2029	€ 4.373,20	€ 3.609,74	€ 763,46	0,82270247	€ 628,10
2030	€ 4.407,68	€ 3.466,15	€ 941,52	0,78352617	€ 737,71
2031	€ 4.441,62	€ 3.327,02	€ 1.114,60	0,7462154	€ 831,73
2032	€ 4.475,03	€ 3.192,27	€ 1.282,76	0,71068133	€ 911,63
2033	€ 4.507,92	€ 3.061,84	€ 1.446,08	0,67683936	€ 978,76
2034	€ 4.540,30	€ 2.935,67	€ 1.604,63	0,64460892	€ 1.034,36
2035	€ 4.572,17	€ 2.813,67	€ 1.758,50	0,61391325	€ 1.079,57
2036	€ 4.603,55	€ 2.695,78	€ 1.907,77	0,58467929	€ 1.115,44
2037	€ 4.634,43	€ 2.581,91	€ 2.052,52	0,55683742	€ 1.142,92
2038	€ 4.664,83	€ 2.472,00	€ 2.192,83	0,53032135	€ 1.162,91
2039	€ 4.694,75	€ 2.365,95	€ 2.328,79	0,50506795	€ 1.176,20
2040	€ 4.724,19	€ 2.263,70	€ 2.460,49	0,4810171	€ 1.183,54
2041	€ 4.753,17	€ 2.165,15	€ 2.588,02	0,45811152	€ 1.185,60
2042	€ 4.781,69	€ 2.070,22	€ 2.711,46	0,43629669	€ 1.183,00
2043	€ 4.809,75	€ 1.978,83	€ 2.830,92	0,41552065	€ 1.176,31
2044	€ 4.837,36	€ 1.890,89	€ 2.946,48	0,39573396	€ 1.166,02
2045	€ 4.864,53	€ 1.806,30	€ 3.058,23	0,37688948	€ 1.152,61
2046	€ 4.891,26	€ 1.724,99	€ 3.166,27	0,35894236	€ 1.136,51
2047	€ 4.917,56	€ 1.646,87	€ 3.270,70	0,34184987	€ 1.118,09
2048	€ 4.943,43	€ 1.571,83	€ 3.371,60	0,32557131	€ 1.097,70
2049	€ 4.968,88	€ 1.499,81	€ 3.469,08	0,31006791	€ 1.075,65
2050	€ 4.993,91	€ 1.430,70	€ 3.563,22	0,29530277	€ 1.052,23
2051	€ 5.018,53	€ 1.364,42	€ 3.654,12	0,28124073	€ 1.027,69
2052	€ 5.042,74	€ 1.300,88	€ 3.741,87	0,26784832	€ 1.002,25
2053	€ 5.066,55	€ 1.239,99	€ 3.826,56	0,25509364	€ 976,13
2054	€ 5.089,96	€ 1.181,68	€ 3.908,29	0,24294632	€ 949,50
2055	€ 5.112,98	€ 1.125,84	€ 3.987,14	0,23137745	€ 922,53
Total	€ 141.639,31	€ 70.520,26	€ 71.119,05		€ 29.250,89

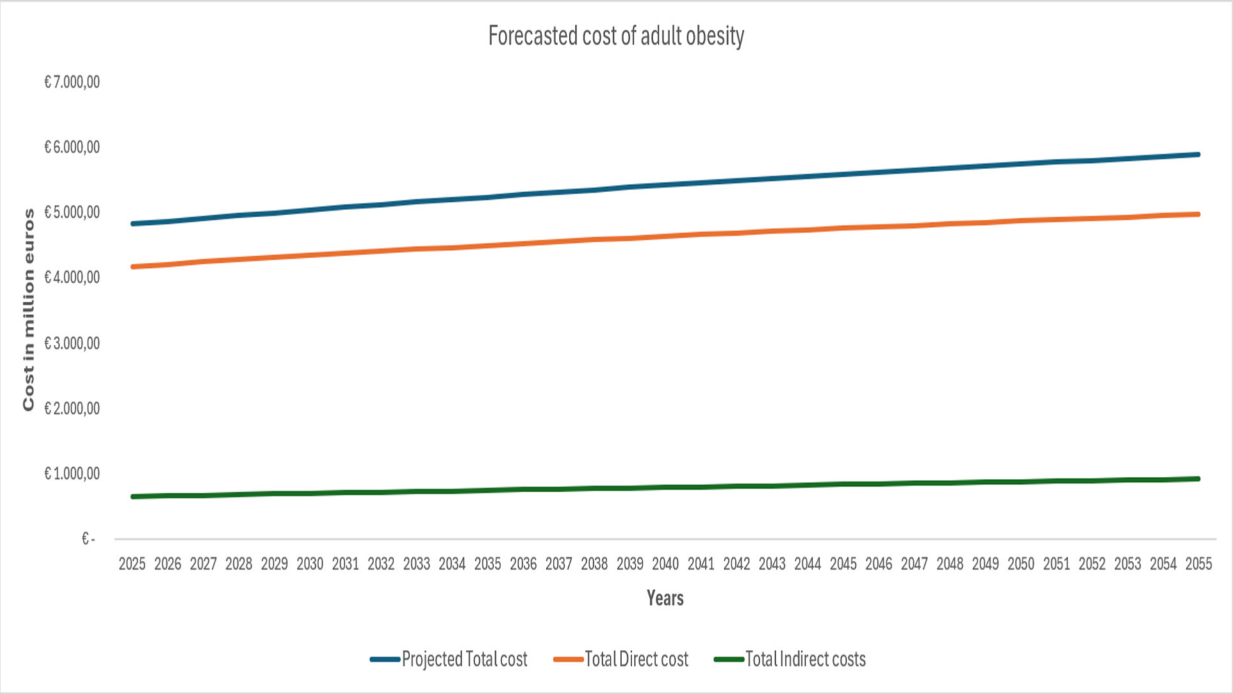

Supplementary Figure S1: Forecasted cost of adult obesity under the future status quo scenario

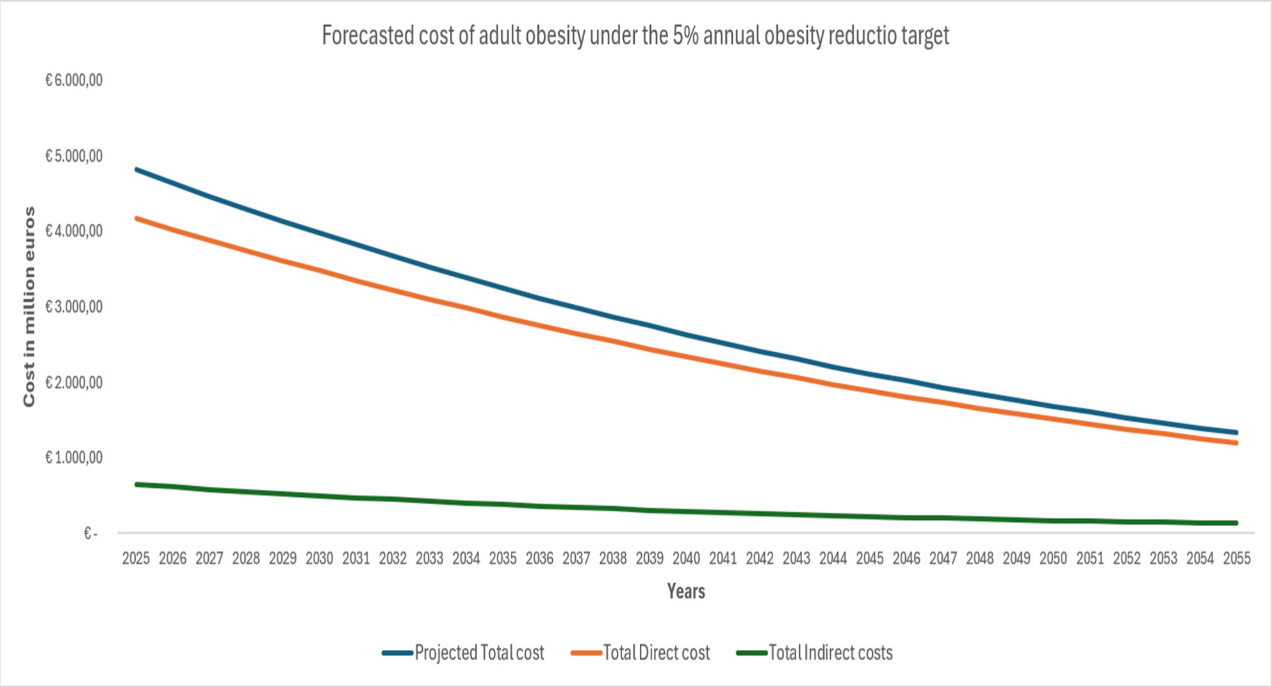

Supplementary Figure S2: Forecasted cost of adult obesity under the 5% annual obesity reduction scenario

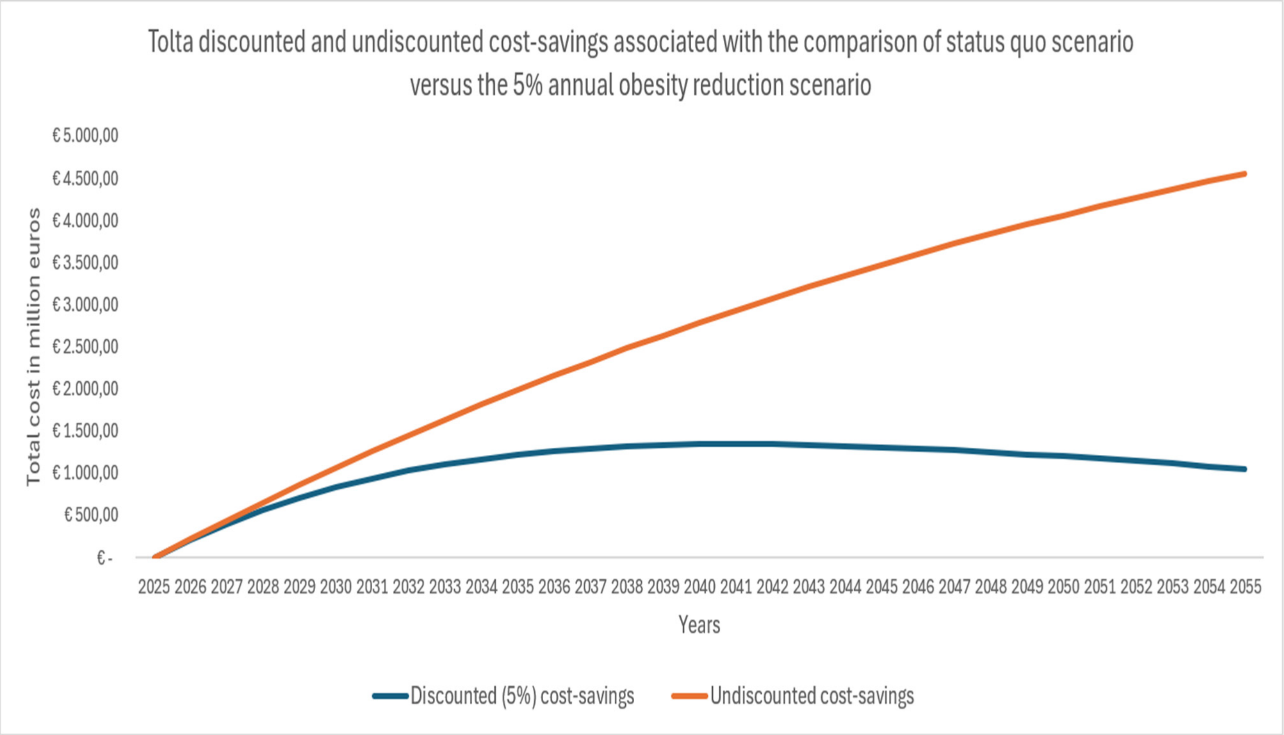

Supplementary Figure S3: Forecasted cost-savings associated with the comparison of the status quo versus the 5% annual obesity reduction scenario
